# Supplementary material for: Control of Intestinal Type 3 Immunity by the Unfolded Protein Response Sensor Inositol-Requiring Enzyme 1α in Antigen-Presenting Cells
Source: Cell Mol Gastroenterol Hepatol. 2026 Sep 2;20(12):101877. doi: 10.1016/j.jcmgh.2026.101877 (PMC13677055; doi:10.1016/j.jcmgh.2026.101877)
Supplement: Supplementary file 1 [file mmc1.pdf]

Supplementary Table I. Antibodies for flow cytometry and cell sorting

Antigen	Fluorophore	Company	Clone	Catalog
XCR1	PE	Biolegend	ZET	148204
CD3	PE-Cy5	Biolegend	145-2C11	100309
CD3	Brillian Violet 510	Biolegend	145-2C11	100353
B220	PE-Cy5	Biolegend	RA3-6B2	103209
B220	Brilliant Violet 510	Biolegend	RA3-6B2	103222
B220	Brilliant Violet 750	Biolegend	RA3-6B2	103239
CD172a	PE-Cy7	Biolegend	P84	144007
CD11c	APC	eBioscience	N418	17-0114-82
CD11c	Brilliant Violet 570	Biolegend	N418	117331
CD11c	PE	Biolegend	N418	117307
CD11c	PE-Cy7	eBioscience	N418	25-0114-82
CD11c	Brilliant Violet 711	Biolegend	N418	117349
Ly6G	Alexa Fluor 700	Biolegend	1A8	127622
MHC II	APC-Cy7	Biolegend	M5/114.15.2	107628
EpCAM	Brilliant Violet 421	Biolegend	G8.8	118225
Ly6C	Brillian Violet 605	Biolegend	HK1.4	128035
NK1.1	Brillian Violet 650	Biolegend	PK136	108736
CD64	Brillian Violet 711	Biolegend	X54-5/7.1	139311
CD64	PerCP-Cy5.5	Biolegend	X54-5/7.1	139307
CD64	PE Dazzle 594	Biolegend	X54-5/7.1	139319
CD11b	APC	Biolegend	M1/70	101211
CD11b	Brilliant Violet 786	Biolegend	M1/70	101243
CD45	PerCPCy5.5	Biolegend	30-F11	103132
CD45	Brillian UltraViolet 395	BD Biosciences	30-F11	564279
CD45	Brillian Violet 480	Invitrogen	30-F11	414-0451-80
CD45	Brilliant Violet 650	Biolegend	30-F11	103151
CD45.2	Alexa Fluor 647	Biolegend	104	109818
F4/80	Biotin	Biolegend	BM8	123105
CD103	PE-Cy7	Biolegend	2E7	121425
CD103	PerCP-eFluor 710	Invitrogen	2E7	121425
CD8	eFluor 450	eBioscience	53-6.7	48-0081-80
CX3CR1	Brilliant Violet 510	Biolegend	SA011F11	149015
CD4	PE Dazzle 594	Biolegend	GK1.5	100455

CD4	APC-Cy7	Tonbo Bioscience	GK1.5	25-0041
KLRG1	PerCP-Cy5.5	Biolegend	2F1/KLGR1	138418
CD127	PE-Cy7	Biolegend	A7R34	135014
CD90.2	APC-Cy7	Biolegend	30-H12	105328
CD90.2	FITC	Biolegend	30-H12	105305
TCRγδ	PerCPCy5.5	Biolegend	GL3	118117
IFNg	PE	eBioscience	XMG1.2	12-7311-82
IL-17	eFluor 660	Invitrogen	eBio17B7	50-7177-82
IL-17	PE-Cy7	Invitrogen	eBio17B7	25-7177-80
IL-17	BV605	BD Biosciences	TC11-18H10	564169
IL-22	PE	Biolegend	Poly5164	516404
RORγt	APC	Invitrogen	AFKJS-9	17-6988-80
RORγt	RB705	BD Biosciences	Q31-378	570259
FOXP3	PE-Cy7	Invitrogen	FJK-16s	25-5773-82
CXCR6	PE	Biolegend	SA051D1	151103
SLAMF6	APC	Biolegend	330-AJ	134609
SLAMF6	Pacific Blue	Biolegend	330-AJ	134608
PDL1	PE	Invitrogen	MIH5	12-5982-82
CD40	APC	Biolegend	3/23.	124611
CD86	PE-Cy7	Biolegend	GL-1	105013
Plet1	Purified	Depreter et al.[81]	1D4	
Goat anti-rat IgG	PE	Biolegend	Poly4054	405416
Goat anti-rat IgG	Alexa Fluor 647	Biolegend	Poly4054	405406
IL-22BP	APC	R&D Systems	Polyclonal	IC2376A
Isotype Sheep IgG (isotype IL-22BP)	APC	R&D Systems	Polyclonal	IC016A
CD16/32 (FcBlock)	Purified	Biolegend	93	101302
Streptavidin	PE-Cy7	Biolegend		405206
Streptavidin	BD Horizon BUV737	BD Biosciences		564293

Supplementary Table II. Primer for q-PCR.

**Gene target**	**Forward 5' → 3'**	**Reverse 5' → 3'**
L27	GCCAAGCGATCCAAGATCAA	GCTGGGTCCCTGAACACATC
Il10	CTTACTGACTGGCATGAGGATCA	GCAGCTCTAGGAGCATGTGG
Tgfb	ACCATGCCAACTTCTGTCTG	CGGGTTGTGTTGGTTGTAGA
Il17a	TTTTCAGCAAGGAATGTGGA	TCCATTGTGGAGGGCAGAC
Il22	ATGAGTTTTTCCCTTATGGGGAC	GCTGGAAGTTGGACACCTCAA
Il6	CACAGAGGATACCACTCCCAACA	TCCACGATTTCCCAGAGAACA
Il1b	AAGCCTCGTGCTGTCGGACC	TGAGGCCCAAGGCCACAGGT
Il23a	CGTATCCAGTGTGAAGATGGTT	CTATCAGGGAGTAGAGCAGGCT
Cxcl1	GACCATGGCTGGGATTCACC	GTGTGGCTATGACTTCGGTT
Cxcl5	TGCGTTGTGTTTGCTTAACCG	CTTCCACCGTAGGGCACTG
Reg3g	ATGCTTCCCCGTATAACCATCA	CGCTATTGAGCACAGATACGAG
Reg3b	ACTCCCTGAAGAATATACCCTCC	CGCTATTGAGCACAGATACGAG
16s rRNA	AGAGTTTGATCMTGGCTCAG	GGMTACCTTGTTACGACTT
Ccl22	TCTTGCTGTGGCAATTCAGA	GAGGGTGACGGATGTAGTCC
Aldh1a1	ATGGTTTAGCAGCAGGACTCTTC	CCAGACATCTTGAATCCACCGAA
Aldh1a2	GACTTGTAGCAGCTGTCTTCACT	TCACCCATTTCTCTCCCATTTCC
Aldh1a3	GAGCAGCAATTTCCTCCCATC	GAGCCGGTGAAGGCTATCT
Il22ra2	ACAACGAGCACTACTTTGTGCAG	CCCCCAGCAGTCAACTTTAT
Cd36	GCGACATGATTAATGGCACAG	GATCCGAACACAGCGTAGATAG
Il27ebi3	CAAGTACCGACTCCGCTACC	GGGTTTCGAGTTCCTGAGGG
Il27p28	GGCTATGTCCACAGCTTTGCT	CGAAGTGTGGTAGCGAGGAA
Ern1 exon 19-20	TGCTGAAACACCCCTTCTTC	GCCTCCTTTTCTATTCGGTCA
Xbp1s	AAGAACACGCTTGGGAATGG	CTGCACCTGCTGCGGAC
Duox2	TGCGCCTGTTACTGTGATTG	AATGGAAAGCAGCAGACAGC
Duoxa2	ACCGCTGCTCATTGTTATCC	AGTGCACAGCCACAATTTCG
Saa1/2	AGTGGCAAAGACCCCAATTA	GGCAGTCCAGGAGGTCTGTA
Muc2	ATGCCCACCTCCTCAAAGAC	GTAGTTTCCGTTGGAACAGTGAA
