# Supplementary material for: Control of Intestinal Type 3 Immunity by the Unfolded Protein Response Sensor Inositol-Requiring Enzyme 1α in Antigen-Presenting Cells
Source: Cell Mol Gastroenterol Hepatol. 2026 Sep 2;20(12):101877. doi: 10.1016/j.jcmgh.2026.101877 (PMC13677055; doi:10.1016/j.jcmgh.2026.101877)
Supplement: Supplementary file 2 [file mmc2.pdf]

cmgh
CELLULAR AND MOLECULAR GASTROENTEROLOGY AND HEPATOLOGY

ORIGINAL RESEARCH

Control of Intestinal Type 3 Immunity by the Unfolded Protein Response Sensor Inositol-Requiring Enzyme 1α in Antigen-Presenting Cells

Dominique Fernández,[1,2,*] Antonia Geisse,[1,2,*] Pamela González-Maldonado,[1,2] Ailiñ Tapia,[1,2] Francisca Gutiérrez-Ballesteros,[1,2] Valentina Ugalde,[3] Felipe Tapia,[1,2] Felipe Flores-Santibañez,[1,2] Takao Iwawaki,[4] Karina Pino-Lagos,[5] Louis Boon,[6] María Rosa Bono,[2] Carolina Prado,[3,7] Rodrigo Pacheco,[3,7] and Fabiola Osorio[1,2]

[1]Laboratorio de Inmunología y Estrés Celular, Núcleo Interdisciplinario de Farmacología e Inmunología, Instituto de Ciencias Biomédicas (ICBM), Facultad de Medicina, Universidad de Chile, Santiago, Chile; [2]Laboratorio de Inmunología, Departamento de Biología, Facultad de Ciencias, Universidad de Chile, Santiago, Chile; [3]Laboratorio de Neuroinmunología, Centro Científico y Tecnológico de Excelencia Ciencia and Vida, Fundación Ciencia and Vida, Santiago, Chile; [4]Division of Cell Medicine, Department of Life Science, Medical Research Institute, Kanazawa Medical University, Uchinada, Ishikawa, Japan; [5]Facultad de Medicina, Centro de Investigación e Innovación Biomédica, Universidad de los Andes, Santiago, Chile; [6]JJP Biologics, Warsaw, Poland; and [7]Facultad de Medicina y Ciencia, Universidad San Sebastián, Santiago, Chile

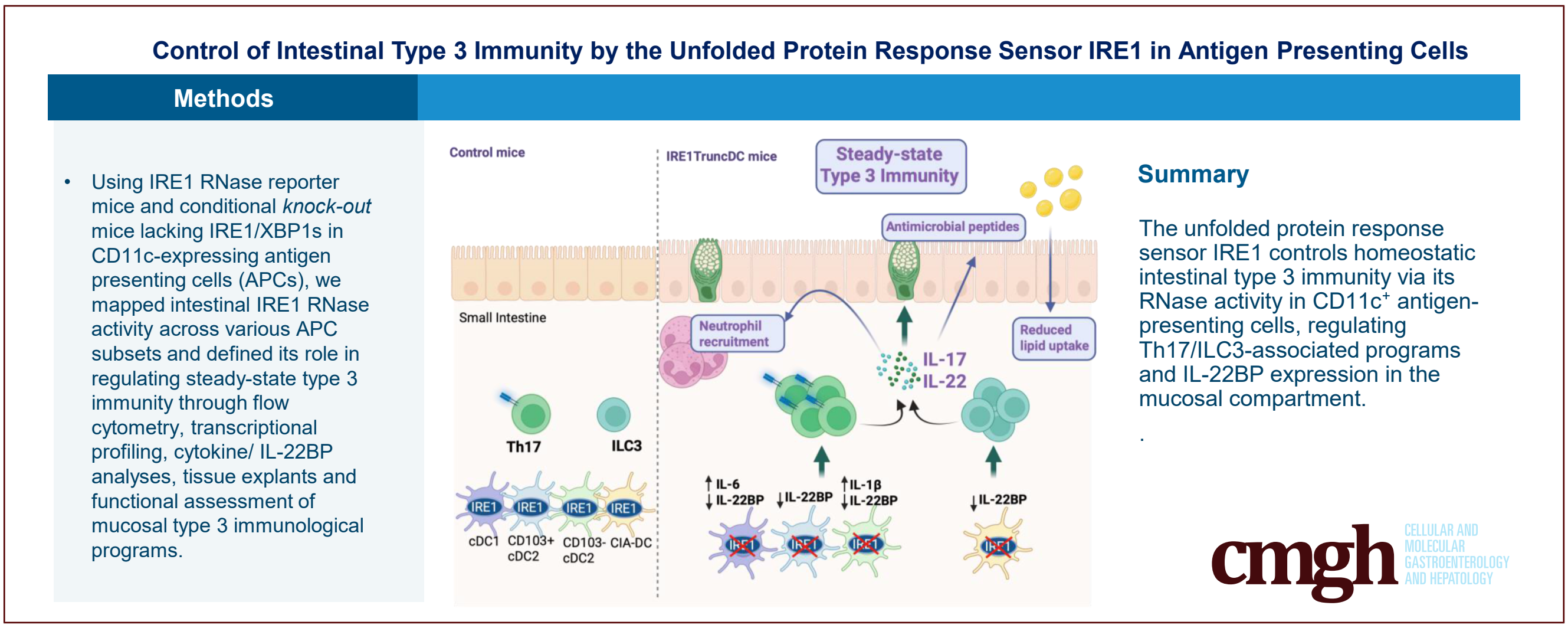

SUMMARY

This study identifies IRE1 RNase activity in intestinal antigen-presenting cells as a critical regulator of type 3 immunity. Through a mechanism independent of XBP1s, IRE1 restrains inflammatory responses and maintains intestinal homeostasis by modulating cytokine expression and interleukin-22 availability.

WHAT YOU NEED TO KNOW

Background: Intestinal immunity depends on balanced antigen-presenting cell function to prevent inflammation. How the stress-response sensor inositol-requiring enzyme 1α controls these cells to maintain immune equilibrium remains insufficiently understood.

Impact: This work identifies inositol-requiring enzyme 1α endoribonuclease as a key regulator of intestinal type 3 immunity, positioning antigen-presenting cell–intrinsic unfolded protein response pathways as promising therapeutic entry points for further studying intestinal inflammatory disorders.

Future Directions: Future studies should evaluate the pharmacologic modulation of inositol-requiring enzyme 1 endoribonuclease activity, define actionable targets of inositol-requiring enzyme 1α activity in intestinal antigen-presenting cell subtypes, and assess the translational potential in patient-derived tissues and preclinical models of intestinal inflammation.

BACKGROUND & AIMS: The unfolded protein response is an essential cellular program maintaining intestinal homeostasis by acting on several cell types. However, whether the unfolded protein response sensor inositol-requiring enzyme 1α acts on dendritic cells and other antigen-presenting cells for shaping intestinal immunity remains poorly defined.

METHODS: Here, we dissect the role of the endoribonuclease domain of the unfolded protein response sensor inositol-requiring enzyme 1α in intestinal CD11c$^+$ antigen-presenting cells, using inositol-requiring enzyme 1α endoribonuclease reporter mice and conditional loss-of-function models for inositol-requiring enzyme 1α and its downstream transcription factor, X-box binding protein 1 spliced.

RESULTS: We identify robust inositol-requiring enzyme 1α endoribonuclease activity in intestinal conventional dendritic cell subsets, dendritic cells associated with crypto patches and isolated lymphoid follicles, and macrophages, independently of microbial signals. Targeted ablation of inositol-requiring enzyme 1α endoribonuclease in the intestinal CD11c$^+$ compartment provoked spontaneous features of type 3 immunity in the small intestine, characterized by increased antimicrobial peptides and neutrophil recruitment, as well as elevated production of interleukin 17 and interleukin 22 by T helper 17 cells and group 3 innate lymphoid cells. This dysregulated activation required inositol-requiring enzyme 1α endoribonuclease but occurred independently of X-box binding protein 1 spliced, implicating regulated inositol-requiring enzyme 1α–dependent decay rather than canonical X-box binding protein 1 spliced signaling. Mechanistically, distinct inositol-requiring enzyme 1α endoribonuclease–deficient intestinal antigen-presenting cell subtypes such as conventional dendritic cells exhibited increased *Il6*, *Il1b*, and *Ccl22* transcript levels, whereas conventional dendritic cells and dendritic cells associated with crypto patches and isolated lymphoid follicles showed marked reductions in interleukin 22BP expression, a profile known to increase interleukin 22 bioavailability and enhance T helper 17 cell–group 3 innate lymphoid cell responses.

CONCLUSIONS: Collectively, our findings uncover inositol-requiring enzyme 1α endoribonuclease in intestinal CD11c$^+$ antigen-presenting cell subtypes as a critical checkpoint restraining type 3 immunity and maintaining intestinal immunity in steady state, highlighting its potential as therapeutic target for intestinal inflammation. *(Cell Mol Gastroenterol Hepatol 2026;20:101877; https://doi.org/10.1016/j.jcmgh.2026.101877)*

Keywords: APCs; Intestine; IRE1; Type 3 Immunity.

The intestine is a dynamic organ crucial for maintaining health by balancing nutritional absorption with the detection of harmful agents. Central to this dual function are antigen-presenting cells (APCs), including dendritic cells (DCs) and macrophages, which are strategically distributed across the intestine. These cells sample environmental antigens for initiation of adaptive immunity or contribute to local responses through crosstalk with resident immune cells, such as innate lymphoid cells (ILCs).[1–4] The intestinal lamina propria harbors several APC subtypes, including conventional type 1 DCs (cDC1, defined as CD103$^+$CD11b$^-$XCR1$^+$ cells) and conventional type 2 DCs (cDC2, identified by CD103$^-$CD11b$^+$ and CD103$^+$CD11b$^+$ profiles),[1,5–9] which orchestrate T-cell tolerance or immunity, depending on the nature of the encountered threat. In addition, intestinal macrophages also support local T-cell responses to commensals and infectious pathogens,[10] whereas more recently identified APC subsets such as APCs expressing the transcription factor retinoid-related orphan receptor γt (RORγt), or DCs residing within crypto patches and isolated lymphoid follicles (termed 'CIA-DCs') promote tolerance to food antigens or control ILC function, respectively.[4,11–19]

A relevant mechanism safeguarding immune responses at mucosal sites is the so-called type 3 immunity, a program commanded by CD4$^+$ T helper 17 (Th17) cells and type 3 ILCs (ILC3s), which protect the intestinal barrier through production of the effector cytokines interleukin (IL)17 and IL22.[1,20,21] This program promotes neutrophil infiltration, expression of antimicrobial peptides and epithelial cell turnover, playing a protective role against infections with extracellular pathogens.[21–23] However, beyond its protective role in infection contexts, exacerbation of type 3 immunity can cause detrimental inflammation and immunopathology.[24] In

*Authors share co-first authorship.

Abbreviations used in this paper: APC, antigen-presenting cell; BSA, bovine serum albumin; CCL2, C-C motif chemokine ligand 2; cDC, conventional dendritic cell; cDC1, conventional type 1 dendritic cell; cDC2, conventional type 2 dendritic cell; CIA-DCs, dendritic cells residing within crypto patches and isolated lymphoid follicles; Ct, cycle threshold; CXCR, CXC chemokine receptor; DC, dendritic cell; ER, endoplasmic reticulum; FACS, fluorescence-activated cell sorting; FBS, fetal bovine serum; FOXP3, forkhead box P3; HBSS, Hank's balanced salt solution; IEC, intestinal epithelial cell; IL, interleukin; ILC, innate lymphoid cell; ILC3, type 3 innate lymphoid cell; IRE1, inositol-requiring enzyme 1α; miRNA, micro RNA; MLN, mesenteric lymph node; mRNA, messenger RNA; NK, natural killer; OVA, ovalbumin; PBS, phosphate-buffered saline; qPCR, quantitative polymerase chain reaction; RIDD, regulated inositol-requiring enzyme 1–dependent decay; RNase, endoribonuclease; RORγt, retinoid-related orphan receptor γt; SI-LP, small intestine lamina propria; SLAMF6, signaling lymphocytic activation molecule family member 6; TGF, transforming growth factor; Th17, T helper 17; UPR, unfolded protein response; Venus FP, Venus fluorescent protein; WT, wild-type; XBP1, X-box binding protein 1; XBP1s, X-box binding protein 1 spliced.

Most current article

© 2026 The Authors. Published by Elsevier Inc. on behalf of American Gastroenterological Association Institute. This is an open access article under the CC BY license (http://creativecommons.org/licenses/by/4.0/).
2352-345X
https://doi.org/10.1016/j.jcmgh.2026.101877

this context, the identification of the APC subtype responsible for regulating intestinal type 3 immunity is a matter of intense research. cDC2s, macrophages, and certain subsets of RORγt^{+} APCs have been shown to instruct intestinal Th17 responses to distinct antigens, highlighting functional redundancy.[7,25–27] Furthermore, the function of ILC3s can also be regulated by DCs, as ILC3 expansion is regulated by CIA-DCs within local lymphoid follicles via production of IL22BP, a soluble IL22 receptor.[4] On mechanistic level, innate signaling pathways linked to Toll-like receptor 5 signaling and regulators of nuclear factor-κB signaling (such as TNFAIP3 and NIK) in DCs have been identified as potential drivers of intestinal Th17 differentiation.[8,28–31] These data indicate that multiple APCs are involved in type 3 immune induction in the gut and that the mechanisms regulating Th17 and ILC3 cell maintenance by APCs remain to be fully understood.

The unfolded protein response (UPR) is a signaling pathway that contributes to intestinal homeostasis by protecting cells from the detrimental effects of endoplasmic reticulum (ER) stress.[32] This cytoprotective response is orchestrated by 3 ER-resident sensors: inositol-requiring enzyme 1α (IRE1), activating transcription factor 6, and PKR-like eIF2α kinase, which play vital roles in cell survival, proliferation, and inflammation.[33–36] IRE1, the most conserved UPR sensor with demonstrated roles in immunity,[33,37,38] possesses serine and threonine kinase domain–engaging proinflammatory signaling pathways,[39–43] and an endoribonuclease (RNase) domain. The RNase domain of IRE1 processes the transcription factor XBP1 spliced (XBP1s), the master regulator of ER biogenesis.[44–46] IRE1 RNase also cleaves additional messenger RNAs (mRNAs) and pre–micro RNAs (miRNAs) bearing a consensus sequence through a process termed 'regulated IRE1-dependent decay' or RIDD.[47,48] Interestingly, XBP1s and RIDD play relevant roles in immunity, metabolism, inflammation, and cell survival.[49–53]

The IRE1–XBP1s axis is a recognized regulator of intestinal homeostasis through roles that extend beyond proteostatic control.[54–57] Mice bearing the selective loss of XBP1s in intestinal epithelial cells (IECs) exhibit Paneth cell impairment, spontaneous enteritis, and Crohn's disease–like ileitis.[58,59] IRE1 activation in IECs also contributes to intestinal tumorigenesis, IgA responses, and Th17 activity via purine metabolism.[54–56] The IRE1–XBP1s axis has also emerged as a regulator of intestinal ILC3 function, influencing mucosal responses.[60] Notably, the relevance of the IRE1–XBP1s axis in intestinal human health is underscored by genetic studies identifying XBP1 as a susceptibility gene for Crohn's disease and ulcerative colitis.[61]

Despite these findings, the relevance of IRE1–XBP1s activation in fine tuning the function of intestinal APCs remains to be defined. Nevertheless, previous reports have shown that cDC1s associated with intestinal tissue exhibit elevated IRE1 RNase activation compared with their counterparts from extraintestinal sites.[62–64] Furthermore, in the small intestine lamina propria (SI-LP), IRE1 RNase activation confers survival to cDC1s undergoing ER stress through mechanisms dependent on RIDD activation and inhibition of protein synthesis.[64] Despite these findings, it remains unclear whether IRE1 RNase activity in intestinal APC subtypes can shape long-lasting mucosal immune responses.[62–64]

Here, we demonstrate that selective ablation of IRE1 RNase in the CD11c-expressing compartment results in spontaneous activation of type 3 immunity in the SI-LP, characterized by increased IL17 and IL22 production, elevated frequencies of Th17 cells, heightened neutrophil infiltration, and enhanced barrier integrity without signs of exacerbated inflammation. Furthermore, IRE1-dependent control of type 3 immunity by CD11c^{+} cells does not require XBP1s, highlighting a role for RIDD in the regulation of intestinal homeostasis. On the functional level, IRE1 RNase regulates the expression of type 3 polarizing factors by diverse APC subtypes; whereas IL6, C-C motif chemokine ligand 2 (CCL-2), and IL1β transcription is increased in cDCs deficient for IRE1 RNase, and the expression of IL22BP is markedly decreased in IRE1-deficient cDC and CIA-DC subtypes. This phenotype is associated with reduced lipid transporter expression and decreased lipid uptake by epithelial cells, recapitulating observations reported in IL22BP-deficient mice. Altogether, these findings highlight the critical role of IRE1 RNase in regulating intestinal type 3 immunity and its potential role as a regulator of lipid metabolic homeostasis in the gut.

Results

Intestinal Antigen-Presenting Cells Exhibit Signs of Inositol-Requiring Enzyme 1α Endoribonuclease Activation

Intestinal cDC1s exhibit heightened IRE1 RNase activity relative to their extraintestinal counterparts.[64] Also, gut resident cells such as goblet cells, IECs, and ILC3s show signs of IRE1–XBP1s activation.[60,61] Thus, we hypothesized that the intestinal milieu promotes IRE1 RNase activation across intestinal immune cells. To this end, we quantified IRE1 RNase activation utilizing the ERAI transgenic mouse strain[65] that reports IRE1 RNase activity through expression of a partial human XBP1 sequence containing the IRE1 splicing sites, fused to Venus fluorescent protein (Venus FP), and validated in multiple reports[63,64,66] (Figure 1*A*). We employed a universal gating strategy of multiparametric flow cytometry (reported in[67]) (Figure 2*A*) to compare the VenusFP signal in a range of innate/adaptive immune cell populations and epithelial cells from the SI-LP. Among SI-LP immune cells, cDC1s expressed high levels of VenusFP, in line with reported findings.[64] CD103^{+} and CD103 cDC2s and intestinal macrophages also showed significant expression of VenusFP (Figure 1*B* and *C*), feature also observed in natural killer (NK) T cells, whereas IECs exhibited background fluorescence (Figure 1*B* and *C*). Additional intestinal immune cells, including monocytes, neutrophils, eosinophils, NK cells, and B and T lymphocytes, did not express VenusFP signal (Figure 1*B* and *C*). Unsupervised t-distributed stochastic neighbor embedding analysis performed on CD45^{+} cells (Figure 1*D* and *E*, Figure 2*D*), revealed that VenusFP expression was mainly restricted to myeloid cell subsets (Figure 1*E*). ILC3s, which are recently reported to activate the IRE1–XBP1s axis,[60]

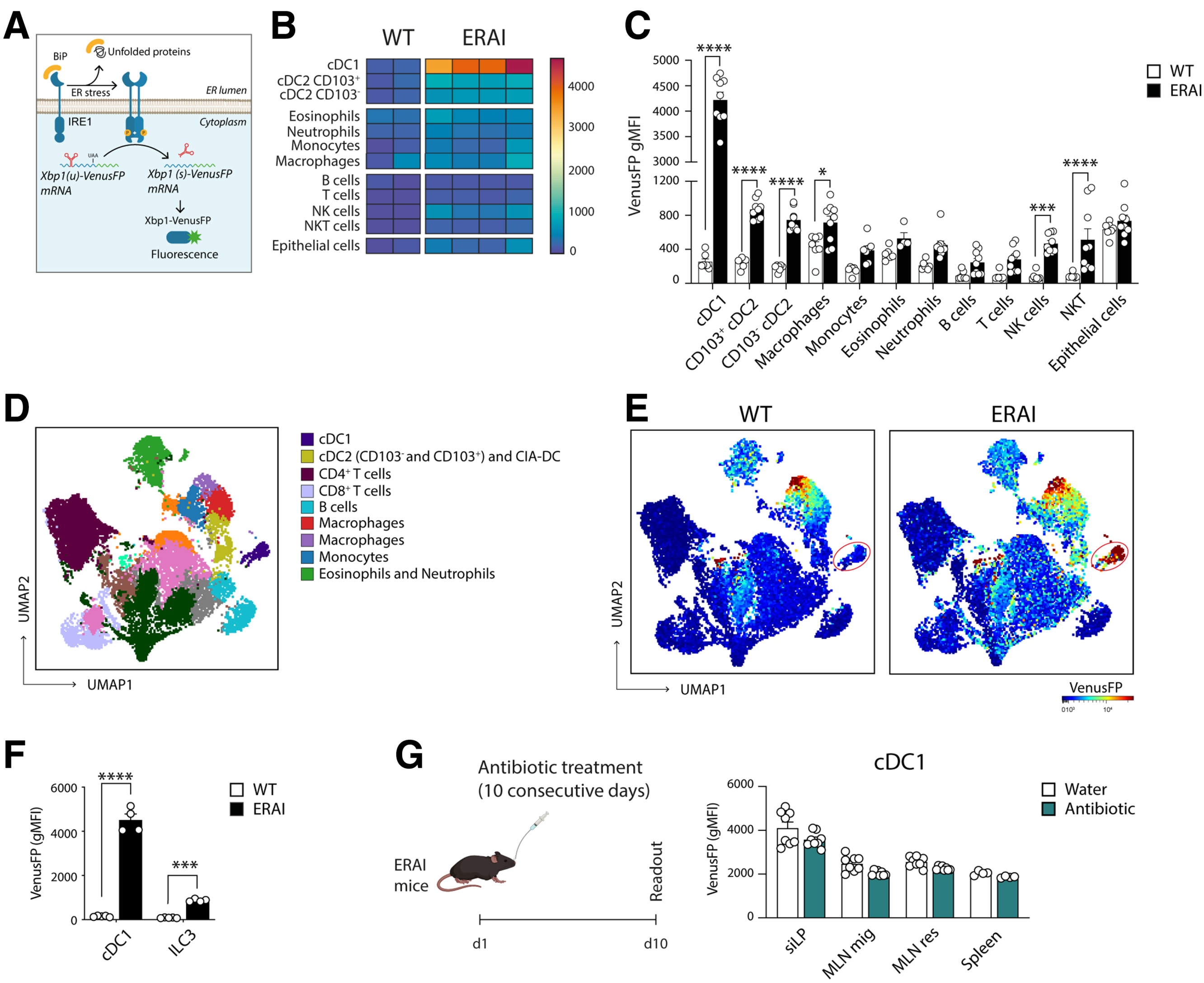

Figure 1. Activation of IRE1 RNase in intestinal immune cells. (*A*) Scheme of the ERAI mouse: under ER stress conditions, IRE1 RNase mediates unconventional splicing of XBP1 mRNA, prompting the translation of XBP1s coupled to VenusFP. (*B*) Heatmap analysis of ERAI VenusFP fluorescence in immune and epithelial cell populations derived from the SI-LP. (*C*) VenusFP fluorescence (gMFI) in immune cells from the SI-LP of ERAI transgenic animals compared with WT mice. n = 9 mice per group, data pooled from 3 independent experiments, mean ± standard error of the mean. *$P < .05$, ***$P < .001$, ****$P < .0001$. (*D*) Uniform Manifold Approximation and Projection (UMAP) of $CD45^+$ immune cells from SI-LP; coloring indicates unsupervised clustering by FlowSOM. (*E*) UMAP representation of VenusFP expression in control (WT) or ERAI mice. The cDC1 cluster is highlighted in a *red circle*. (*F*) VenusFP fluorescence (gMFI) in cDC1s and ILCs from the SI-LP of ERAI transgenic animals compared with WT mice. n = 4–5 mice per group, data pooled from 3 independent experiments; mean ± standard error of the mean. ***$P < .001$, ****$P < .0001$. (*G*) ERAI mice were treated with antibiotics or vehicle via gavage for 10 consecutive days, and VenusFP fluorescence (gMFI) was measured in cDC1s from the SI-LP, migratory MLNs (MLN mig), resident mesenteric lymph nodes (MLN res), and spleen. n = 4–8 mice per group, data pooled from 2 independent experiments. Data show the mean ± standard error of the mean. Data in (*C* and *F*) were analyzed with 2-way analysis of variance with Šídák's multiple comparisons test. Data in (*G*) were analyzed with the Kruskal–Wallis test.

also expressed noticeable VenusFP fluorescence, albeit at lower levels than cDC1s (Figure 1*F*, Figure 2*B* and *C*). Altogether, these data indicate that, in the SI-LP, IRE1 RNase is predominantly active among DC subsets and macrophages, followed by NK, NKT, and ILC3 populations.

In the gut, the IRE1–XBP1s axis is proposed to be induced by multiple triggers, including the microbiota.[68] To determine whether IRE1 RNase activation observed in intestinal DCs is microbiota dependent, we measured VenusFP signal in intestinal cDC1s from ERAI mice treated for 10 days with an antibiotic cocktail or a vehicle control. As shown in Figure 1*G*, VenusFP expression by cDC1s from the SI-LP, mesenteric lymph nodes (MLNs) and spleen remained unaltered upon antibiotic treatment, suggesting that IRE1 RNase activation in DCs does not rely on bacterial colonization.

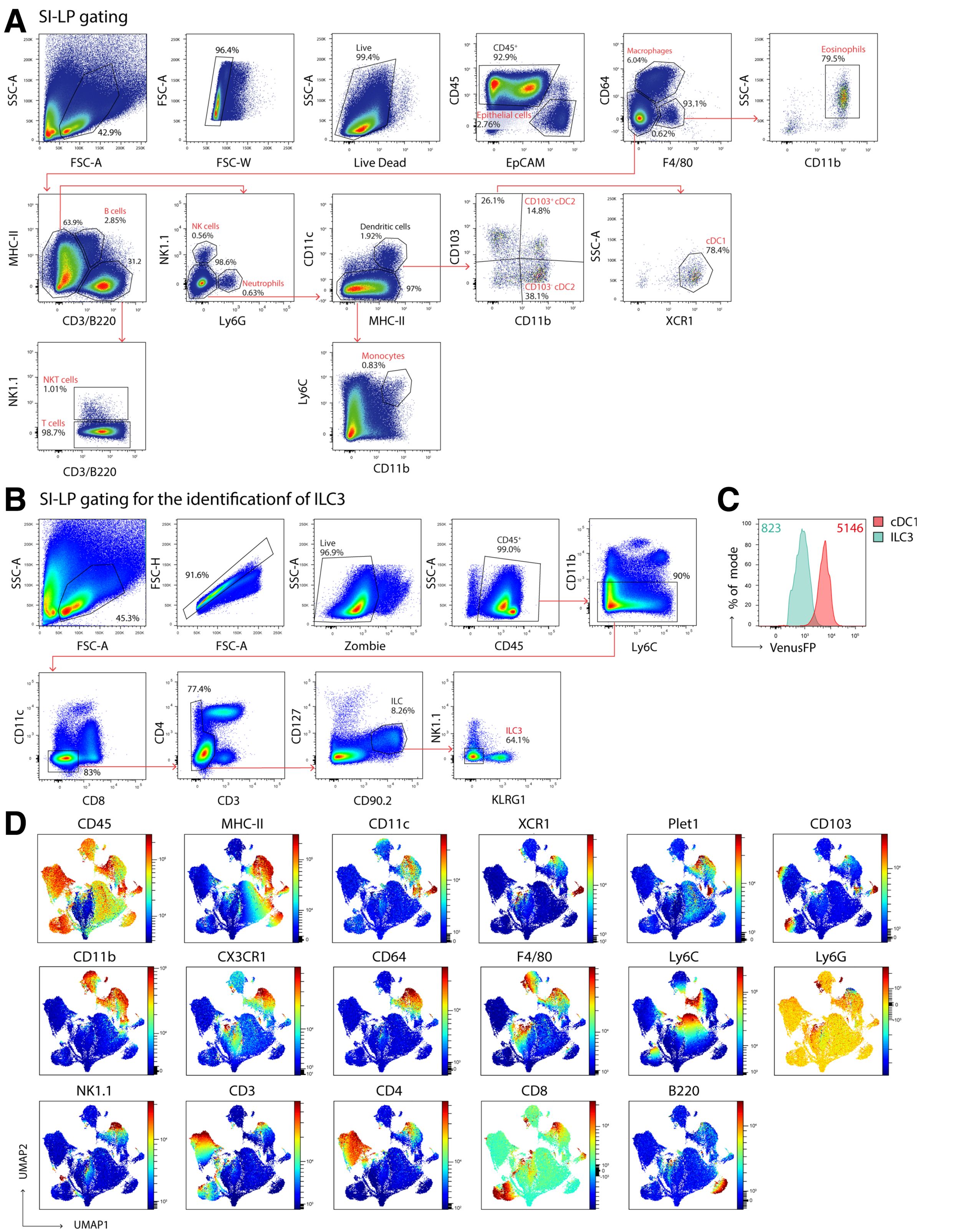

Figure 2. Gating strategy for the identification of immune cells from the SI-LP. (*A*) Gating strategy for cell identification in the SI-LP. (*B*) Gating strategy for the identification of ILC3 from the SI-LP. (*C*) Representative histograms of VenusFP fluorescence (gMFI) within cDC1 and ILC3 cells from ERAI transgenic animals compared with WT mice. (*D*) Uniform Manifold Approximation and Projection (UMAP) accompanying Figure 1*E*. Color gradient shows the expression of the indicated marker.

Spontaneous Activation of Type 3 Immunity in Duodenum of IRE1^{TruncDC} Mice

We sought to investigate the functional relevance of IRE1 RNase activation in APCs for intestinal immune homeostasis. To this end, we crossed the *Itgax*-Cre (CD11c-Cre) mice line[69] with *Ern1* flox/flox mice,[70] which express loxP sites flanking exons 19 and 20 of *Ern1* gene (coding IRE1). In the intestine, CD11c is expressed by multiple APC subsets including cDCs, macrophages, CIA-DCs, and a fraction of RORγt^{+} APCs.[4,7,13,14,19,71,72] Accordingly, our CD11c–Cre strategy interrogates the CD11c^{+} compartment in aggregate. The generated mice line, named ‘IRE1^{TruncDC}’ mice, expresses a truncated version of IRE1 lacking the RNase domain in the CD11c^{+} compartment.[63] Experimental controls correspond to littermates *Ern1* flox/flox lacking Cre recombinase (‘IRE1^{WT}’). In agreement with previous work,[64] IRE1^{TruncDC} mice showed normal composition of CD103^{+} and CD103^{-} cDC2s (Figures 3*A* and 5*A–C*), although some experimental variability was observed. Also, as reported, IRE1^{TruncDC} mice exhibited decreased frequencies of cDC1s

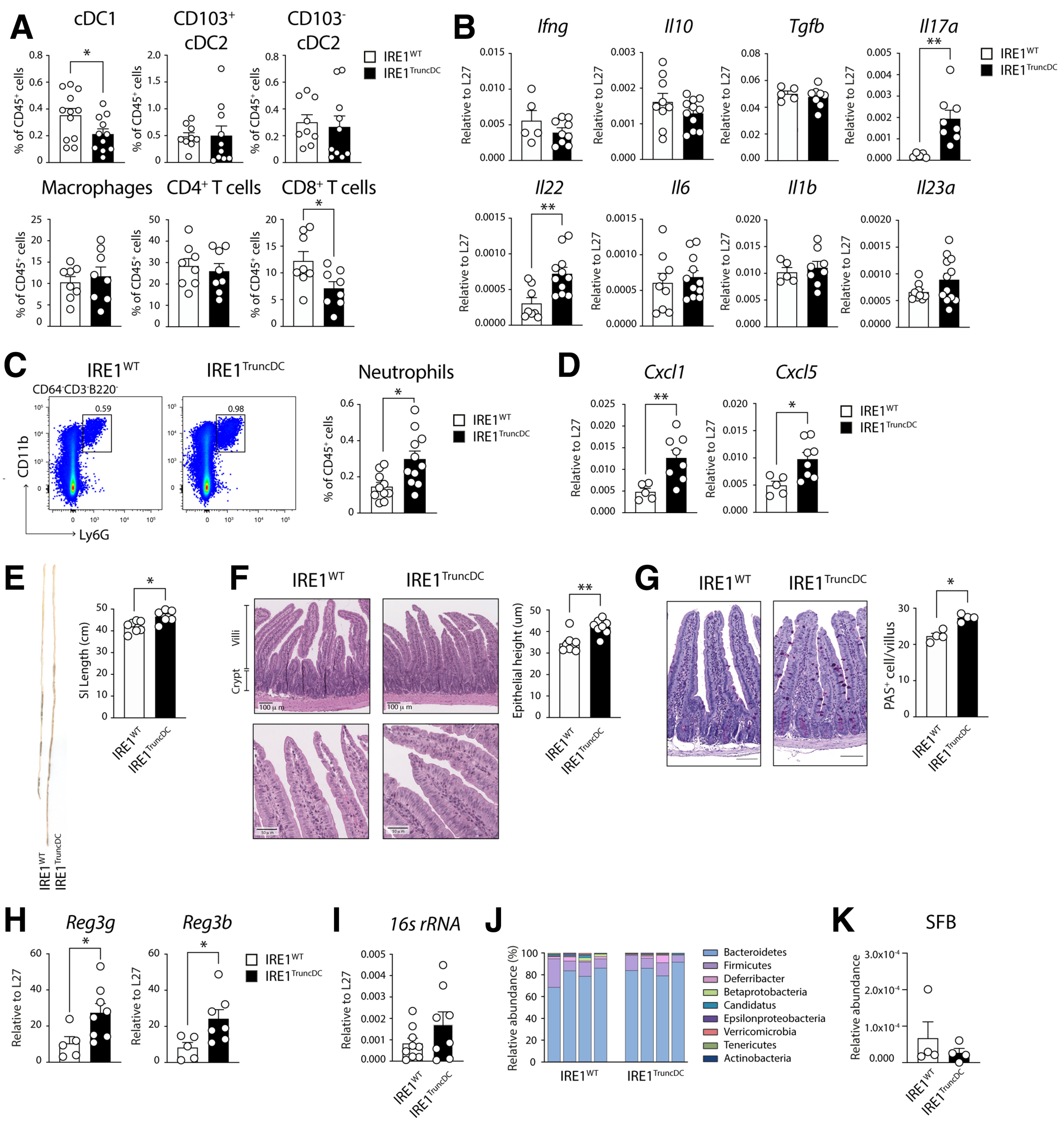

in the SI-LP and in the migratory compartment of the MLNs (Figures 3*A* and 5*A*–*C*), consistent with the reported role of IRE1 in controlling cDC1 homeostasis. However, cDC1s and $CD103^+$ and $CD103^-$ cDC2 from $IRE1^{TruncDC}$ mice exhibited functional abrogation of IRE1 RNase activity in the SI-LP, evidenced by reduced levels of *Xbp1s* in these cells compared with their control counterparts (Figure 5*D*). Macrophages, monocytes, plasmacytoid DCs, B cells, and $CD4^+$ T cells displayed similar frequencies between $IRE1^{TruncDC}$ and control mice, although a mild reduction in $CD8^+$ T cells was noticed in conditional deficient animals (Figure 3*A*, Figure 5*E*). Furthermore, comparable total cellularity was found in SI-LP preparations between conditional knockout mice and controls (Figure 5*I*).

Next, we collected RNA from the small intestine of $IRE1^{TruncDC}$ and $IRE1^{WT}$ control littermates and assessed a range of pro- and anti-inflammatory cytokines using quantitative polymerase chain reaction (qPCR). Duodenal tissues from $IRE1^{TruncDC}$ mice express normal levels of the proinflammatory factors interferon (IFN)-γ, IL6, IL1β, IL23, and the anti-inflammatory cytokines IL10 and transforming growth factor (TGF)-β compared with their control counterparts (Figure 3*B*). In contrast, a marked accumulation of IL17 and IL22 transcripts were found in the duodenum from $IRE1^{TruncDC}$ mice relative to controls (Figure 3*B*). Considering that IL17 and IL22 are hallmark cytokines of type 3 immunity, we investigated whether the small intestine of $IRE1^{TruncDC}$ mice exhibited tissue signs of this immune program. Flow cytometry analysis revealed increased neutrophil accumulation in the SI-LP from $IRE1^{TruncDC}$ mice (Figure 3*C*), accompanied by elevated transcription of the neutrophil chemoattractants C-X-C motif chemokine ligand 1 and C-X-C motif chemokine ligand 5 (Figure 3*D*). Also, the small intestine of $IRE1^{TruncDC}$ mice was consistently larger than those of the $IRE1^{WT}$ control littermates (Figure 3*E*) and showed signs of epithelial hypertrophy (Figure 3*F*). These features were accompanied by a significant increase in goblet cell numbers (Figure 3*G*) and increased levels of transcripts encoding antimicrobial proteins Reg3g and Reg3b, which are known barrier-associated epithelial programs engaged upon type 3 immune activation (Figure 3*H*). Altogether, these data indicate that IRE1 RNase loss in the $CD11c^+$ compartment leads to spontaneous intestinal type 3 immune activation.

Next, we sought to investigate whether the phenotypes observed in $IRE1^{TruncDC}$ mice are attributed to variations in the microbiota. Quantification of 16s ribosomal RNA levels of predominant bacterial genera revealed no significant differences between transgenic and control mice, and similar levels of the Th17 inducer segmented filamentous bacteria were found between transgenic and control mice (Figure 3*I*–*K*). These data suggest that the immunologic variations observed between IRE1-deficient and -sufficient animals are unlikely to be driven by microbiota changes.

$IRE1^{TruncDC}$ Mice Exhibit Accumulation of T Helper 17 Cells and Type 3 Innate Lymphoid Cells in the Small Intestine Lamina Propria

To further understand the immune phenotype of $IRE1^{TruncDC}$ mice, cytokine measurements were carried out in duodenum, jejunum, and ileum explants of $IRE1^{TruncDC}$ and control mice. Analysis revealed that $IRE1^{TruncDC}$ mice showed a significant increase of secreted IL17 in the duodenum and jejunum, highlighting that regulation of IRE1 RNase is restricted to the upper-mid small intestine (Figure 4*A*). Notably, differences in cytokine secretion were only observed for IL17, as other effector cytokines, including IFN-γ, IL4, IL10, and IL2, remained unaltered between $IRE1^{TruncDC}$ and $IRE1^{WT}$ control mice, confirming selective type 3 immune activation without influencing type 1 or type 2 immune programs (Figure 4*A*).

Next, we sought to investigate whether the induction of type 3 immunity observed in $IRE1^{TruncDC}$ mice is coordinated by Th17 cells and/or ILC3s. Flow cytometry analysis revealed that $IRE1^{TruncDC}$ mice display marked accumulation of IL17-producing $CD4^+$ cells in the SI-LP (Figure 4*B*). These Th17 cells were primarily IL17 single producers, although some IL17 and IFN-γ double producers were detected (Figure 4*B*). In the gut, Th17 cells can be subdivided into signaling lymphocytic activation molecule

Figure 3. (***See previous page***). **Spontaneous activation of type 3 immunity in $IRE1^{TruncDC}$ mice.** (*A*) Percentage of intestinal DCs, macrophages, and $CD4^+$ and $CD8^+$ T cells within the $CD45^+$ compartment from SI-LP of WT and $IRE1^{TruncDC}$ mice. n = 8–12 mice per group; data pooled from 2–3 independent experiments; mean ± standard error of the mean (SEM). **P* < .05. (*B*) Relative abundance of cytokine mRNA in the duodenum of WT and $IRE1^{TruncDC}$ mice determined by RT-PCR. n = 5–12 mice per group; data pooled from 3 independent experiments; mean ± SEM. ***P* < .01. (*C*) Representative flow cytometry plots and quantification of intestinal neutrophils within the $CD45^+$ cell compartment from the SI-LP of WT and $IRE1^{TruncDC}$ mice. n = 10–11 mice per group; data pooled from 5 independent experiments; mean ± SEM. (*D*) Relative abundance of the chemokines *Cxcl1* and *Cxcl5* in duodenum of WT and $IRE1^{TruncDC}$ mice determined by RT-PCR. n = 5–8 mice per group; data pooled from 3 independent experiments; mean ± SEM. ***P* < .01. (*E*) Small intestine length measurement from WT and $IRE1^{TruncDC}$ mice. n = 6–7 mice per group; mean ± SEM. * *P* < .05. (*F*) Representative histologic images of duodenal tissue from WT and $IRE1^{TruncDC}$ mice stained with hematoxylin and eosin (H&E), with quantification of epithelial height. n = 8 mice per group; mean ± SEM. ***P* < .01. (*G*) Representative histologic images of duodenal tissue from WT and $IRE1^{TruncDC}$ mice stained with Periodic acid-Schiff (PAS) and quantification of goblet cells (PAS^+ cells along the villus length). n = 4 mice per group; mean ± SEM. ***P* < .01. (*H*) Relative abundance of antimicrobial peptides in the duodenum of WT and $IRE1^{TruncDC}$ mice determined by RT-PCR. n = 5–8 mice per group; data pooled from 3 independent experiments; mean ± SEM. **P* < .05. (*I*) Relative abundance of *16s* ribosomal RNA (rRNA) in duodenal tissue in the duodenal tissue of WT and $IRE1^{TruncDC}$ mice. n = 8–9 mice per group; data pooled from 3 independent experiments, mean ± SEM. (*J* and *K*) Relative abundance of different bacterial phyla was determined by *16s* rRNA gene RT-PCR from fecal pellets. n = 4 mice per group; mean ± SEM. All data were analyzed with the Mann–Whitney test.

family member 6 (SLAMF6)- and CXC chemokine receptor (CXCR)6-positive populations, which bear stem-like homeostatic properties or effector–pathogenic roles, respectively.[73] Whereas Th17 cells from control mice exhibit a balanced proportion of CXCR6^{+} SLAMF6^{+} and CXCR6^{+} SLAMF6^{-} cells, counterparts from IRE1^{TruncDC} mice exhibit a bias towards the CXCR6^{+}SLAMF6^{-} population (Figure 4*C*). Furthermore, frequencies of IL22-producing CD4^{+} T cells were also increased in the SI-LP of IRE1^{TruncDC} mice without influencing Foxp3^{+} regulatory T cell frequencies (Figure 4*D* and *E*).

Next, we sought to investigate whether Th17 expansion also occurred in tissues outside the small intestine. Whereas Th17 frequencies in spleen were comparable between IRE1^{TruncDC} and IRE1^{WT} control mice, Th17 accumulation was also noticed in MLNs from IRE1^{TruncDC} mice, albeit to a lower extent than in the SI-LP (Figure 5*F* and *G*). These data indicate that IRE1 RNase in CD11c^{+} APCs selectively regulates Th17 accumulation in the intestinal tissue and associated draining lymph nodes.

Given their central role in mucosal type 3 immunity, we assessed the ILC3 compartment in the SI-LP of IRE1^{TruncDC}

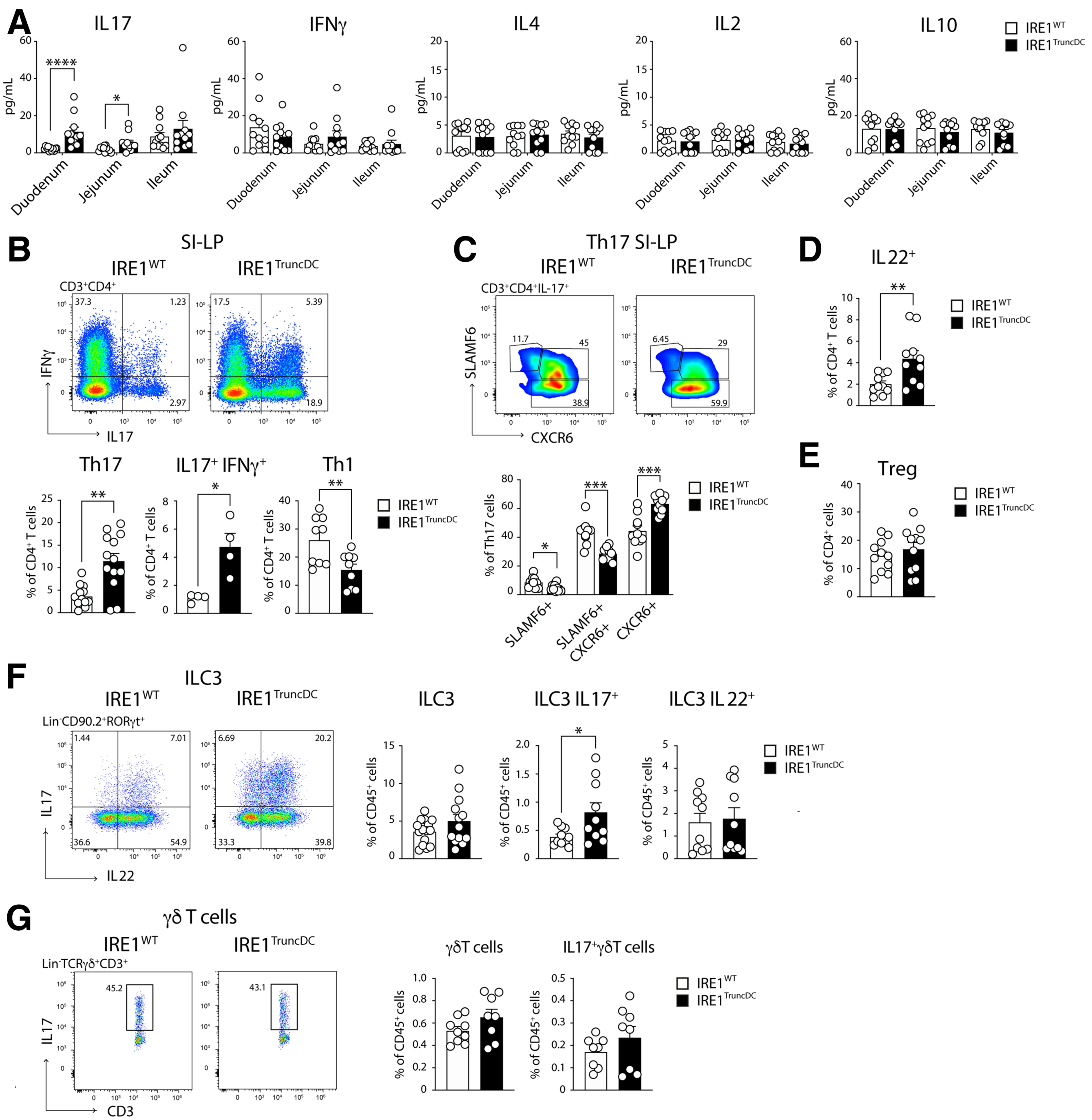

mice. Mirroring the phenotype observed in Th17 cells, the SI-LP of IRE1^{TruncDC} mice exhibited significant increase of IL17-producing ILC3s, whereas the total frequencies of ILC3 and IL22 production on a per-cell basis remained unchanged (Figure 4*F*). By contrast, IL17 production by intestinal $\gamma\delta$ T cells was comparable between IRE1^{TruncDC} and control mice (Figure 4*G*), indicating that not all IL17-producing populations are impacted by IRE1 deficiency in CD11c^{+} cells. Collectively, these findings demonstrate that loss of IRE1 RNase in CD11c^{+} cells selectively expands intestinal Th17 and ILC3s, resulting in elevated accumulation of IL17 and IL22 in the SI-LP of IRE1^{TruncDC} mice.

Inositol-Requiring Enzyme 1α Endoribonuclease in CD11c^{+} Cells Regulates Intestinal Type 3 Immunity Independently of X-Box Binding Protein 1 Spliced Function

Next, we aimed to elucidate the mechanisms underlying the initiation of type 3 immunity in IRE1^{TruncDC} mice. IRE1 RNase can exert its functions via the XBP1s transcription factor or by RNA decay through the RIDD pathway. To determine whether XBP1s or RIDD accounts for the increased IL17 and IL22 responses, we crossed the *Itgax* Cre mice line with Xbp1fl/fl animals, which lack the exon 2 of Xbp1s gene, resulting in loss of the XBP1s in CD11c^{+} cells.[74] We referred to these animals as 'XBP1$^{\Delta DC}$'.[63,64,66] Experimental controls correspond to littermates *Xbp1* flox/flox lacking Cre recombinase ('XBP1^{WT}'). XBP1$^{\Delta DC}$ mice display a normal composition of cDC subsets in the SI-LP, as reported[64] (Figure 6*A*). XBP1s deletion in cDC1s induces compensatory activation of the RIDD pathway[63,64]; thereby, to validate effective XBP1s deletion, we assessed the surface expression of CD11c, a surrogate RIDD marker.[62] CD11c forms a heterodimer with CD18 (*Itgb2*), a bona-fide IRE1 substrate, which, upon RIDD mediated degradation, prevents CD11c from reaching the cell surface, a parameter that can be assessed by flow cytometry[63,64] (Figure 7*A* and *B*). SI-LP cDC1s from XBP1$^{\Delta DC}$ mice exhibited a significant reduction in CD11c surface levels, confirming effective XBP1s ablation (Figure 7*B*).

Next, we functionally evaluated whether XBP1$^{\Delta DC}$ mice also exhibit activation of intestinal type 3 immunity. Data depicted in Figure 6*B* and *C* revealed that the SI-LP of XBP1$^{\Delta DC}$ mice expresses comparable mRNA levels of IL22 and IL17 relative to control littermates without neutrophil accumulation (Figure 6*A* and *B*). In contrast to the results observed in IRE1^{TruncDC} mice, XBP1$^{\Delta DC}$ mice exhibit reduced Th17 frequencies in the SI-LP relative to XBP1^{WT} controls (Figure 6*C*) and histologic hematoxylin and eosin analysis of the duodenum of XBP1$^{\Delta DC}$ mice revealed no signs of epithelial cell hypertrophy (Figure 6*D*). Collectively, these data indicate that IRE1 RNase and XBP1s play divergent roles in the induction of intestinal Th17 cells and suggest that the spontaneous activation of intestinal type 3 immunity is attributed to IRE1 RNase-dependent, XBP1s-independent mechanisms operating in gut-associated CD11c^{+} cells.

IRE1^{TruncDC} Mice Exhibit Normal Frequencies and Function of Retinoid-Related Orphan Receptor γt^{+} Antigen-Presenting Cells

We aimed to identify the APC driving type 3 immunity in IRE1^{TruncDC} mice. We first investigated whether IRE1 RNase deficiency alters phenotype and function of intestinal RORγt^{+} APCs, given their strong association with mucosal immune regulation.[11–15,17] An adapted flow cytometry strategy from[15,19] revealed that the frequencies of SI-LP ILC3–major histocompatibility complex class II^{+} and RORγt^{+} DCs were comparable between IRE1^{TruncDC} and control mice (Figure 8*A*). These subsets were also similarly represented in other tissues such as the MLNs and the spleen from control and conditional knockout mice (Figure 8*B* and *C*), indicating that IRE1 RNase activity does not regulate ILC3–major histocompatibility complex class II^{+} and RORγt^{+} DC frequencies.

Functionally, RORγt^{+} APCs selectively control the homeostasis and function of intestinal RORγt^{+} forkhead box P3 (FOXP3)$^{+}$ double-positive cells, which are involved in promoting tolerance to food and microbiota antigens.[11–15,17] Analysis of RORγt and FOXP3 expression within the CD4^{+} T cell compartment revealed significant expansion of RORγt^{+}FOXP3^{-} but not RORγt^{+}FOXP3^{+} or RORγt^{-}FOXP3^{+} T cells in the SI-LP of IRE1^{TruncDC} mice (Figure 8*D*). Thus, we

Figure 4. (***See previous page***). **Th17 accumulation of IRE1^{TruncDC} mice is selective to the intestine.** (*A*) Cytokine production by different intestinal segments of WT and IRE1^{TruncDC} mice cultured for 24 hours. Cytokine production was measured in the supernatant by cytometric bead array (CBA). n = 3–4 mice per group; data pooled from 3 independent experiments, mean ± standard error of the mean (SEM). *$P < .05$, ****$P < .0001$. (*B*) Representative flow cytometry plots and quantification of intestinal IL17^{+}, IL17^{+}IFNg^{+} and IFNg^{+} producers within the CD4^{+} T cell compartment from the SI-LP of WT and IRE1^{TruncDC} mice. n = 4–13 mice per group; data pooled from 3 independent experiments; mean ± SEM. *$P < .05$, **$P < .01$, ***$P < .001$. (*C*) Representative flow cytometry and quantification of SLAMF6 and CXCR6 expression within the Th17 compartment from the SI-LP of WT and IRE1^{TruncDC} mice. n = 10–11 mice per group; data pooled from 5 independent experiments, mean ± SEM. *$P < .05$, ***$P < .001$. (*D*) Quantification of intestinal IL22 within the CD4^{+} T cell compartment from the SI-LP of WT and IRE1^{TruncDC} mice. n = 10 mice per group; data pooled from 4 independent experiments; mean ± SEM. **$P < .01$. (*E*) Percentage of intestinal regulatory T cells (Tregs) (CD4^{+} Foxp3^{+}) within the CD45^{+} immune cell compartment from the SI-LP of WT and IRE1^{TruncDC} mice. n = 11 mice per group; data pooled from 4–5 independent experiments; mean ± SEM. (*F*) Representative flow cytometry and quantification of IL17 and IL22 expression within the ILC3 (Lin(CD11b, CD11c, B220, CD3, CD4)$^{-}$ CD90.2^{+} RORγt^{+}) compartment from the SI-LP of WT and IRE1^{TruncDC} mice. n = 10–15 mice per group; data pooled from 4–5 independent experiments; mean ± SEM. *$P < .05$. (*G*) Representative flow cytometry and quantification of $\gamma\delta$T cells and IL17 $\gamma\delta$T cells (Lin(CD64, CD8, B220, NK1.1)$^{-}$ TCR$\gamma\delta^{+}$ CD3^{+}) within the CD45^{+} immune cell compartment from the SI-LP of WT and IRE1^{TruncDC} mice. n = 8–9 mice per group; data pooled from 4 independent experiments. All data were analyzed with the Mann–Whitney test.

conclude that IRE1 deletion does not regulate canonical functions of RORγt^{+} APCs. These findings align with the phenotype recently reported for the conditional deletion of IRE1 RNase in the *Rorc*+ compartment, which decreases IL17 production by ILC3s,[60] contrasting with the phenotype observed for the IRE1^{TruncDC} mice displayed here. Collectively, these findings suggest that IRE1 RNase has distinct roles in *Rorc*+ vs CD11c^{+} compartments.

Inositol-Requiring Enzyme 1α Endoribonuclease Loss Increases Expression of T Helper 17 Polarizing Factors by Conventional Dendritic Cell Subtypes

cDC subtypes can prime intestinal Th17 immunity.[7,25,28,29] We evaluated whether IRE1 RNase loss predisposes cDCs for type 3 immune activation. We assessed expression of surface immunoregulatory molecules

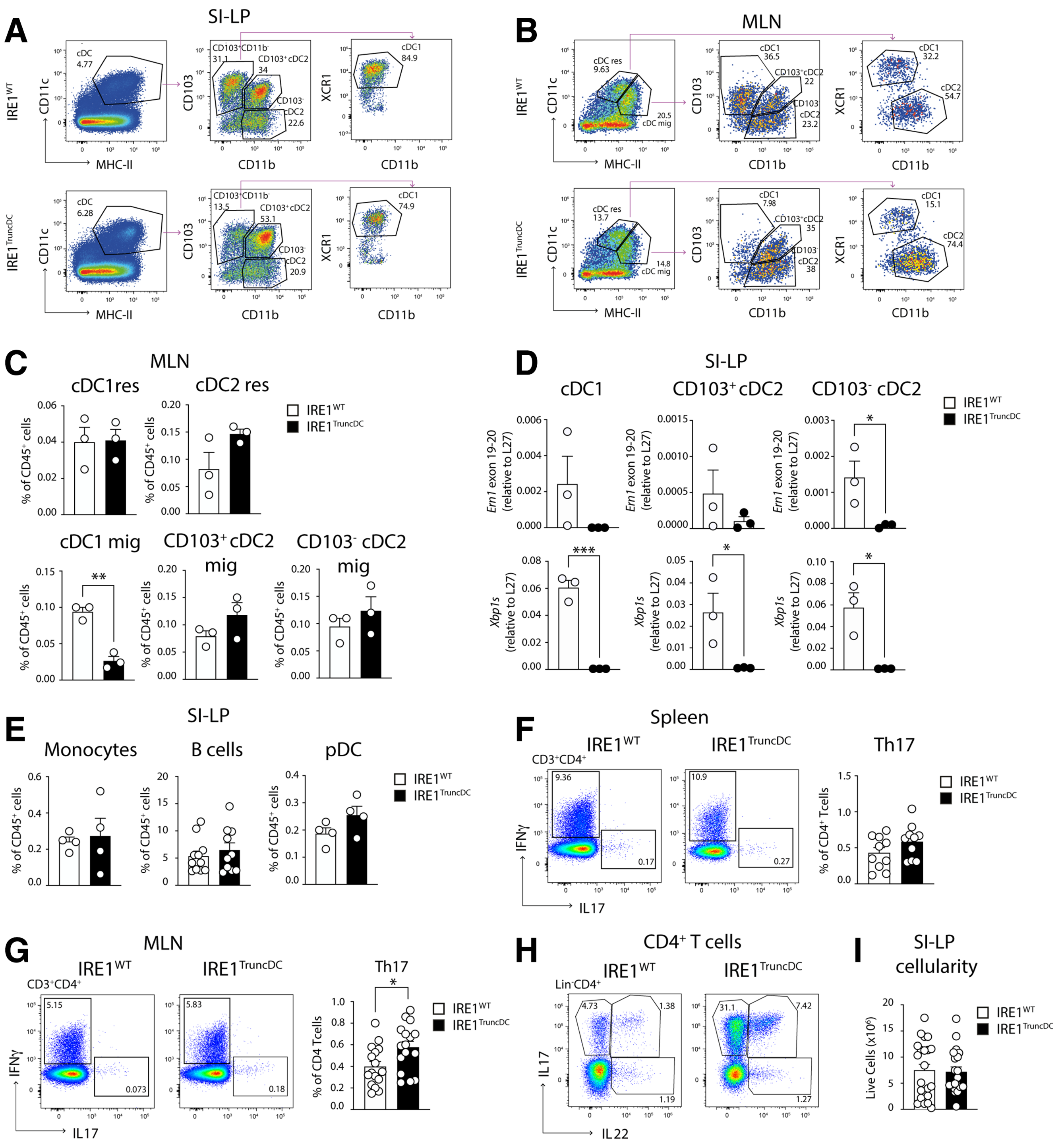

programmed death-ligand 1, CD80, and CD86, which were comparable between cDC1s (Figure 9*A*) and cDC2s (Figure 9*B*) from IRE1^{TruncDC} and control littermates. We next interrogated the antigen presentation capacity of IRE1^{TruncDC} mice upon transfer of CD4^{+} T cells from the transgenic OT-II mice, (which express a TCR recognizing a peptide derived from ovalbumin [OVA]), followed by OVA administration through an intragastrical route (Figure 9*C*). Whereas OT-II T cells exhibit similar proliferative capacity in the MLNs and Peyer Patches of IRE1^{TruncDC} and control mice, a mild but significant proliferative reduction was noticed in the SI-LP of conditional knockout animals (Figure 9*C*). These data suggest that IRE1^{TruncDC} mice display unaltered initial antigen recognition by CD4^{+} T cells in lymph nodes but show moderate impairment in intestinal tissue accumulation, expansion, or survival.

Next, we evaluated whether IRE1 RNase deficiency regulates the expression of Th17 polarizing factors by cDCs. To this end, we sorted cDC1a and CD103^{+} and CD103^{-} cDC2s from the SI-LP of IRE1^{TruncDC} and control mice and quantified cytokines involved in type 3 immune induction, including IL6, TGF-β, IL23, IL1β and CCL22.[75,76] Analysis revealed that cDC1s and CD103^{+}cDC2s from IRE1^{TruncDC} mice displayed elevated levels of IL6 mRNA (Figure 9*D*). In addition, TGF-β mRNA remained unaltered between IRE1^{TruncDC} and IRE1^{WT} control mice, whereas IL23(p19) transcript showed a trend toward increase in cDC1s from IRE1^{TruncDC} mice (Figure 9*D*). Notably, additional factors boosting Th17 cell development/recruitment such as IL1β mRNA and CCL22, were also upregulated in the CD103^{-} cDC2 subset from IRE1^{TruncDC} mice (Figure 9*D*). In addition, the expression of retinaldehyde dehydrogenase mRNA, a key enzyme involved in retinoic acid metabolism for regulatory T cell generation,[77] was significantly reduced in cDC1s from IRE1^{TruncDC} mice (Figure 9*E*). These data indicate that IRE1-deficient intestinal cDCs are reprogrammed to induce the expression of factors promoting Th17 cell induction and recruitment to the gut.

Interleukin22 Binding Protein Expression Is Regulated by Inositol-Requiring Enzyme 1α Endoribonuclease in Intestinal Dendritic Cells

We next focused on studying the mechanisms accounting for the expansion of ILC3 in IRE1^{TruncDC} mice. A circuit regulating ILC3 homeostasis is controlled by CIA-DCs, a specific subset residing in crypto patches and isolated lymphoid follicles.[4] CIA-DCs express CD11c and regulate ILC3 homeostasis through production of IL22BP (also known as IL22RA2), a soluble decoy receptor that blocks bioactive IL22, regulating ILC3 hubs.[4,78,79] IL22BP is also produced by cDCs, emerging as a relevant control of IL22-mediated responses.[77] Expression analysis revealed a significant reduction in *Il22ra2* levels in duodenal tissue from IRE1^{TruncDC} mice relative to controls (Figure 10*A*). Furthermore, IRE1-deficient cDC1 and CD103^{+} and CD103^{-} cDC2 subsets also exhibited reduced expression of *Il22ra2* compared with their control counterparts, with statistical significance noticed in the CD103^{-} cDC2 subset (Figure 10*B*).

We next sought to assess CIA-DC function. These cells are contained within the CD103- cDC2 gate and can be identified by placenta expressed transcript 1 expression.[4,80] We interrogated whether CIA-DCs induce IRE1 RNase activity. Using ERAI mice, we observed that CIA-DCs express high levels of VenusFP, comparable to CD103^{+} and CD103^{-} cDC2s (Figure 10*C*). In addition, CIA-DCs frequencies were similarly represented between IRE1^{TruncDC} and control mice (Figure 10*D*), indicating that IRE1 does not control CIA-DC differentiation and homing into the gut. Next, we analyzed expression of IL22BP on protein levels using flow cytometry (Figure 10*E*). Notably, analysis revealed that CIA-DCs, alongside cDC1s and CD103^{+} and CD103^{-} cDC2s from IRE1^{TruncDC} mice expressed significantly decreased levels of IL22BP relative to controls (Figure 10*E*). Decreased IL22BP expression is expected to increase IL22 bioavailability, resulting in the induction of IL22 target genes associated with the type 3 immune program.[81] Indeed, in addition to the increased expression of Reg3g and Reg3b, which are IL22-dependent genes (Figure 3*H*), we also observed an increased expression of Saa1/2 and Muc2 (which depend on IL22 signaling[82–85]), in the duodenal tissue of IRE1^{TruncDC} mice. Altogether, these results demonstrate that IRE1 RNase deletion results in the broad reduction of IL22BP production by multiple APC subsets with a concomitant increase in the expression of IL22-dependent genes in the SI-LP.

IRE1^{TruncDC} Mice Exhibit Altered Lipid Uptake and Reduced Expression of CD36

Activation of intestinal type 3 immunity can impact physiological responses on the organ level. It is reported

Figure 5. (***See previous page***). **Validation and analysis of IRE1^{TruncDC} mice.** (*A*) Gating strategy for the identification of DCs from SI-LP of WT and IRE1^{TruncDC} mice. (*B*) Gating strategy and (*C*) quantification of DCs in mesenteric lymph nodes of WT and IRE1^{TruncDC} mice. n = 3 mice per group; mean ± standard error of the mean (SEM). $^{**}P < .01$. (*D*) Validation of Cre-mediated exon recombination by the measurement of the relative abundance of *exon 19-20* of *Ern1* and *Xbp1s* in DCs sorted from the SI-LP of WT and IRE1^{TruncDC} mice by RT-PCR. n = 3 mice per group; mean ± SEM. $^{*}P < .05$. (*E*) Percentage of intestinal monocytes, B cells, and plasmacytoid DCs (pDCs) within the CD45^{+} immune cell compartment from the SI-LP of WT and IRE1^{TruncDC} mice. n = 4–8 mice per group; data pooled from 2–3 independent experiments; mean ± SEM. (*F*) Representative flow cytometry plots and quantification of Th17 in the spleen of WT and IRE1^{TruncDC} mice. n = 10 mice per group; data pooled from 3 independent experiments; mean ± SEM. (*G*) Representative flow cytometry plots and quantification of Th17 in the mesenteric lymph node of WT and IRE1^{TruncDC} mice. n = 16 mice per group; data pooled from 5 independent experiments; mean ± SEM. (*H*) Representative flow cytometry plot for the identification of IL17+, IL22+, and double-positive IL17^{+} IL22^{+} cells within CD4 T cells from the small intestine lamina propria of WT and IRE1^{TruncDC} mice. (*I*) SI-LP cellularity obtained after percoll. n = 20–22 mice per group; data pooled from 8 independent experiments; mean ± SEM. Data in (*C* and *D*) were analyzed with unpaired *t* test with Welch's correction. Data in (*E–G* and *I*) were analyzed with the Mann–Whitney test. MHC-II, major histocompatibility complex class II.

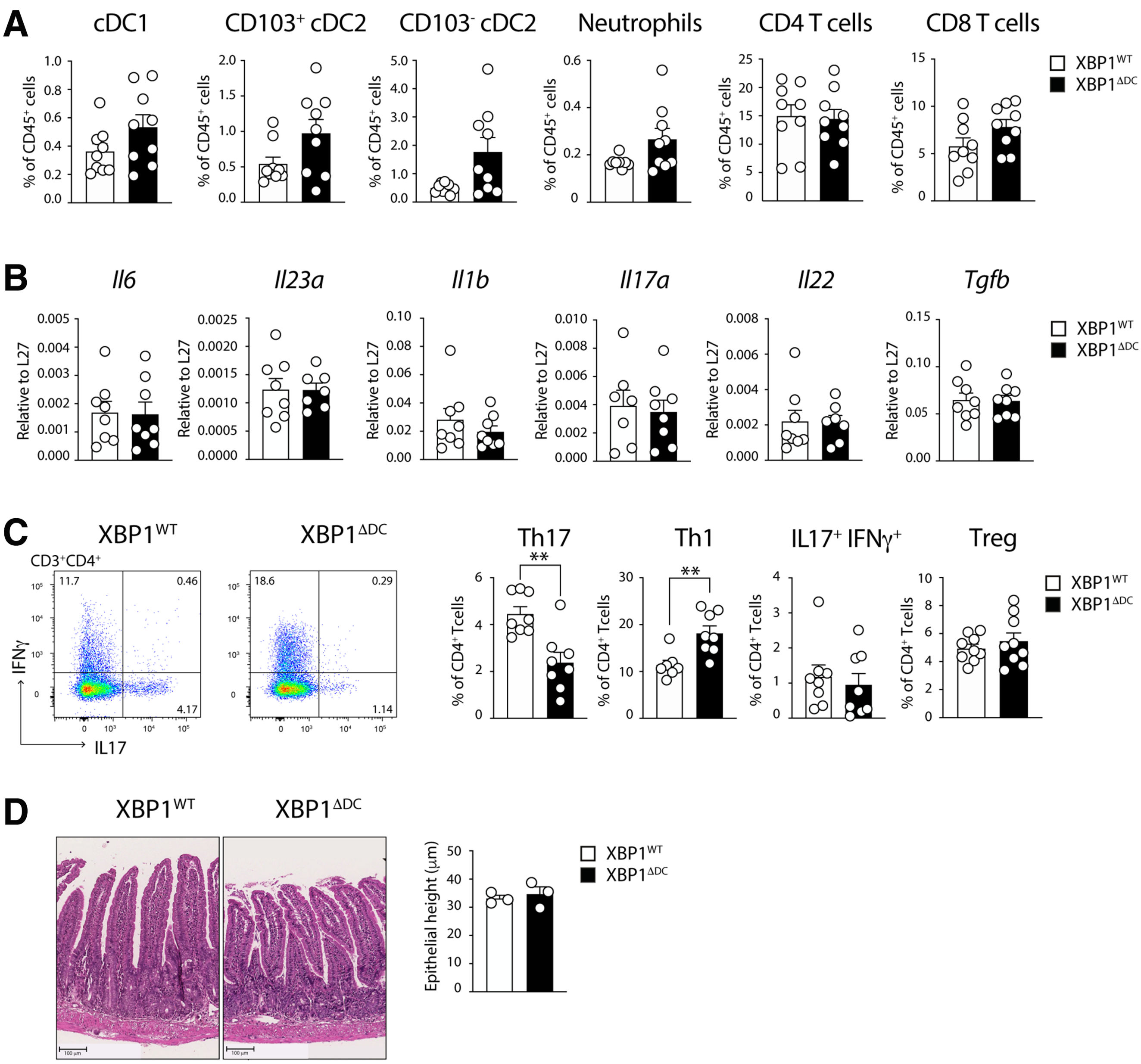

Figure 6. IRE1 RNase in CD11c$^+$ cells control Th17 accumulation by XBP1s-independent mechanisms. (*A*) Percentage of intestinal DCs, neutrophils, CD4$^+$ T cells, and CD8$^+$ T cells within the CD45$^+$ immune cell compartment from the SI-LP of WT and XBP1$^{\Delta DC}$ mice. n = 9 mice per group; data from 4 independent experiment; mean ± standard error of the mean (SEM). *$P < .05$. (*B*) Relative abundance of different cytokines within the duodenum of WT and XBP1$^{\Delta DC}$ mice determined by RT-PCR. n = 7–8 mice per group; data pooled from 2 independent experiments; mean ± SEM. (*C*) Representative flow cytometry plots and quantification of intestinal Th17, IL17$^+$IFNg$^+$, Th1, and regulatory T cells (Tregs) within the CD4$^+$ T cell compartment from SI-LP of WT and XBP1$^{\Delta DC}$ mice. n = 8–9 mice per group; data pooled from 3–4 independent experiments; mean ± SEM. **$P < .01$. (*D*) Representative histologic images of duodenal tissue WT and XBP1$^{\Delta DC}$ mice stained with hematoxylin and eosin (H&E), with quantification of epithelial height. n = 3 mice per group; mean ± SEM. All data were analyzed with the Mann–Whitney test.

that mice models of unrestrained ILC3 expansion, induced by the deletion of adaptive immunity or upon IL22BP deficiency, exhibit impaired lipid metabolism and decreased intestinal lipid transporter expression.[4,86] Thus, considering our findings, we investigated whether IRE1^{TruncDC} mice exhibit alterations in intestinal lipid uptake. We observed that *Cd36* mRNA expression levels were consistently reduced in the duodenum and jejunum of IRE1^{TruncDC} mice compared with their control littermates, aligning with observations in IL22BP knockout mice[4] (Figure 11*A*). Furthermore, lipid uptake experiments in intestinal epithelial cells using fluorescently labeled bodipy revealed decreased lipid absorption by epithelial cells derived from IRE1^{TruncDC} mice relative to controls

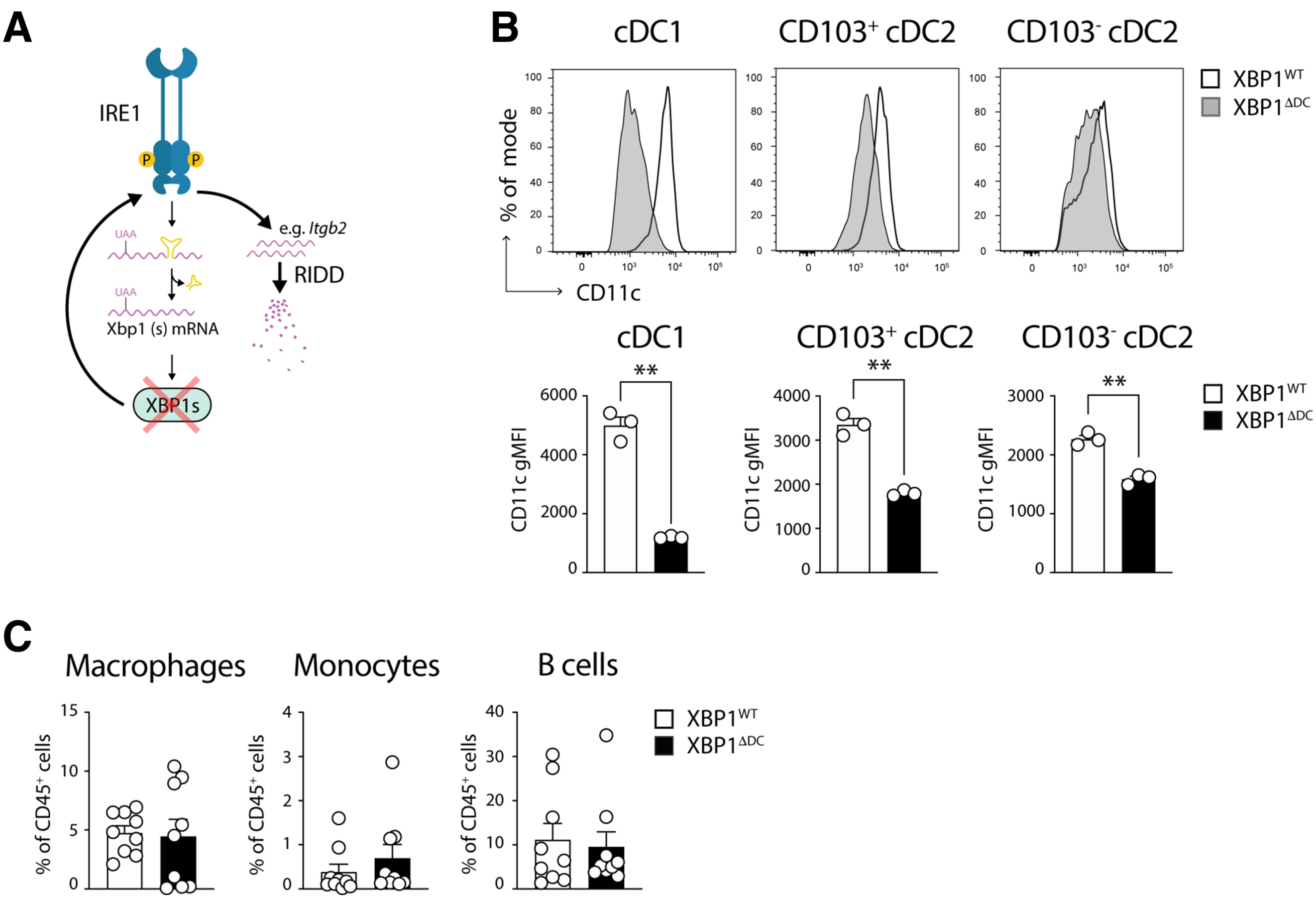

Figure 7. Characterization of XBP1$^{\Delta DC}$ mouse. (*A*) Schematic representation of XBP1$^{\Delta DC}$ mouse. (*B*) Quantification of CD11c expression (gMFI) in DCs from SI-LP of WT and XBP1$^{\Delta DC}$. n = 3 mice per group; data from 1 independent experiment, mean ± standard error of the mean. $^{**}P < .01$. (*C*) Percentage of intestinal macrophages, monocytes and B cells within the CD45$^+$ immune cell compartment from SI-LP of WT and XBP1$^{\Delta DC}$ mice. n = 9 mice per group; data from 4 independent experiments; mean ± standard error of the mean. Data in (*B*) were analyzed with the unpaired *t* test with Welch's correction. Data in (*C*) were analyzed with the Mann–Whitney test.

(Figure 11*B* and *C*). Interestingly, the differences were more prominently noticed in epithelial cells derived from duodenal and jejunal areas, in line with the duodenal accumulation of IL17 noticed in IRE1^{TruncDC} mice (Figure 11*A*). These results suggest that IRE1 RNase in the CD11c-compartment controls lipid absorption by IECs, recapitulating lipid uptake features associated with unrestrained ILC3 responses and IL22BP loss in the gut.[4,86]

Finally, ER stress in IECs also exacerbates Th17 differentiation in the gut, by mechanisms dependent on reactive oxygen species production and purine metabolites generated by the DUOX2–DUOXA2 system.[57] Interestingly, we observed that expression of transcripts coding DUOX–DUOXA2 enzymes were significantly elevated in IECs from IRE1^{TruncDC} mice relative to control cells, highlighting tight reprogramming of the intestinal milieu IRE1^{TruncDC} mice (Figure 11*D*). Notably, these findings are not due to IECs being directly targeted by the CD11c-driven Cre recombinase, as these cells do not delete the exon 19 and 20 of the *Ern1* gene (data not shown). These findings indicate that IRE1 RNase deletion in CD11c$^+$ potentiates intestinal type 3 immunity and organ function via direct reprogramming of intestinal APC subtypes, and in-trans mechanisms operating on IECs.

Discussion

The IRE1–XBP1s axis of the UPR is increasingly recognized as a central regulator of intestinal homeostasis, by acting on multiple cell types including IECs, Paneth cells, and ILC3s.[60,61,87,88] Here, we uncovered an unappreciated role of IRE1 RNase in CD11c$^+$ APCs as a key determinant of intestinal immune balance. Our findings build upon previous findings demonstrating that IRE1 RNase is markedly active in intestinal cDC1s and partially active in cDC2s,[64] but extends these observations to additional APCs such as CIA-DCs and intestinal macrophages. Notably, IRE1 activation occurs even in antibiotic presence, suggesting that nonbacterial, endogenous, or nutritional cues may sustain its activity under homeostatic conditions.

A novel aspect derived from this work is the functional consequence of IRE1 activity in CD11c-APCs, leading to spontaneous activation of intestinal type 3 immunity, and accompanied by Th17 expansion, elevated IL17 and IL22

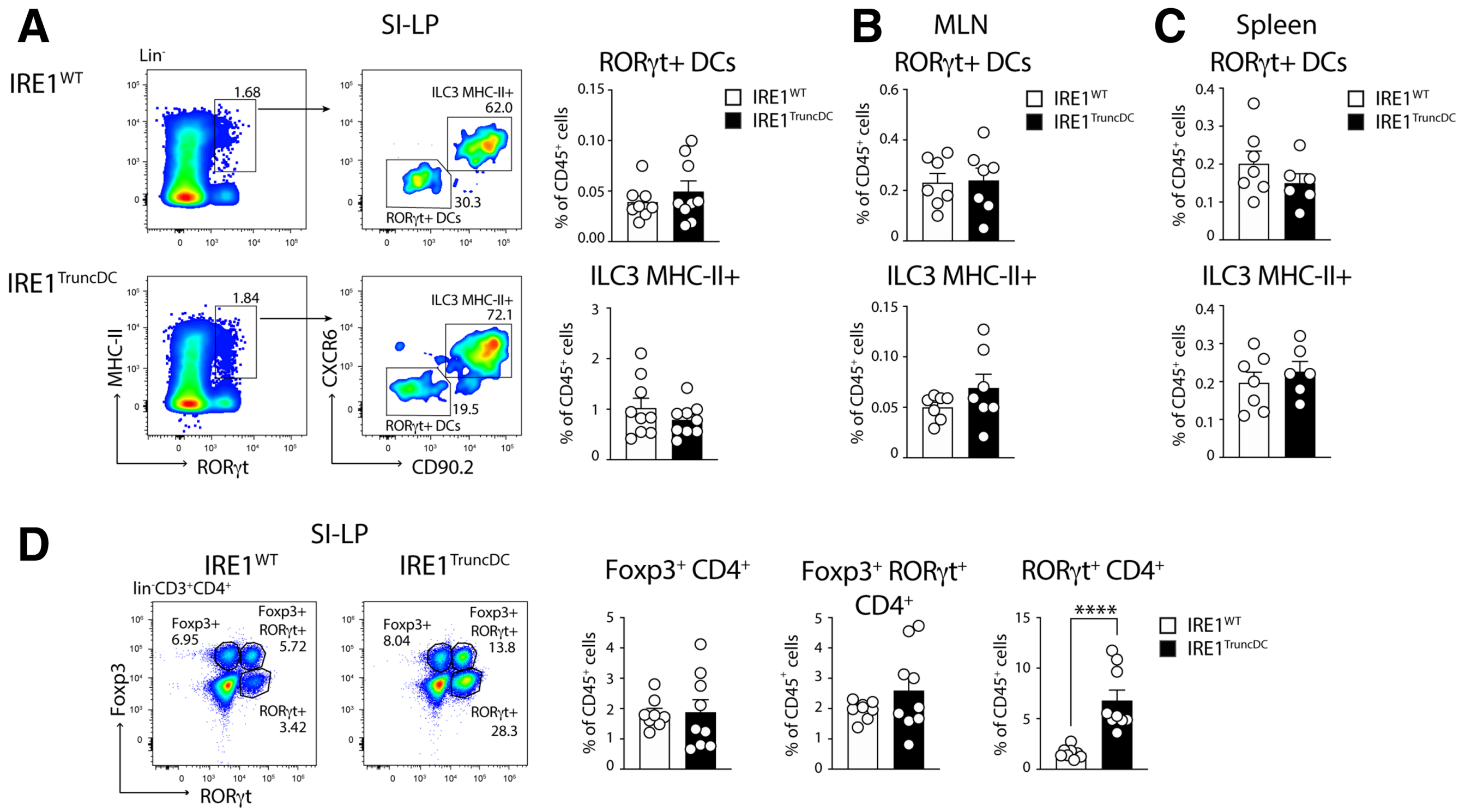

Figure 8. IRE1^{TruncDC} mice exhibit normal frequencies of RORγt$^+$ APCs. (*A–C*) Representative flow cytometry plots and quantification of RORγt$^+$ DCs and ILC3 MHCII, major histocompatibility complex class II (MHC-II)$^+$ in the SI-LP (*A*), MLNs (*B*), and spleen (*C*) within the CD45$^+$ compartment of WT and IRE1^{TruncDC} mice. RORγt$^+$ DCs were defined as Lin(CD64, Ly6C, NK1.1, CD3, B220)$^-$ MHC-II$^+$ RORγt$^+$ CXCR6$^-$ CD90.2$^-$ and ILC3 MHC-II+ were defined as Lin(CD64, Ly6C, NK1.1, CD3, B220)$^-$ MHC-II$^+$ RORγt$^+$ CXCR6$^+$ CD90.2$^+$. n = 6–9 mice per group; data pooled from 4 independent experiments; mean ± standard error of the mean. (*D*) Representative flow cytometry plots and quantification of Foxp3$^+$ CD4$^+$, Foxp3$^+$ RORγt$^+$ CD4$^+$ and RORγt$^+$ CD4$^+$ (Lin(CD64, CD8, B220, NK1.1)$^-$ CD3$^+$) within the CD45$^+$ immune cell compartment from the SI-LP of WT and IRE1^{TruncDC} mice. n = 8-9 mice per group; data pooled from 4 independent experiments; mean ± standard error of the mean. $^{**}P < .01$. All data were analyzed with the Mann–Whitney test.

production, neutrophil recruitment, antimicrobial peptides, and epithelial reprogramming. These findings demonstrate that, in basal conditions, the RNase domain of IRE1 in APCs actively controls Th17 and ILC3 function to maintain intestinal immune balance. The selective accumulation of Th17 cells in the small intestine and MLNs, with no corresponding increases in the spleen, suggests that IRE1 RNase in CD11c$^+$ cells regulates the local maintenance and expansion of IL17$^+$ CD4$^+$ T cells within the intestinal milieu. However, it is relevant to note that the epithelial program of IRE1^{TruncDC} mice was associated with barrier protection without overt inflammation. Furthermore, the activation of type 3 immunity observed in the small intestine of IRE1^{TruncDC} mice appears to be selective rather than associated with a global expansion of immune cells, as total immune cell numbers did not differ between control and conditional-deficient animals. A limitation of this study is the relatively small sample size in the analysis of ILC3 (n = 10 per group), which was lower than the number estimated a priori to achieve 80% power. Nevertheless, the observed large effect size and statistically significant between-group difference in IL17 production support the findings, whereas validation in a larger cohort is warranted. On the other hand, expression of DUOX–DUOX2 transcripts in IECs suggests tight epithelial reprogramming as a consequence of IRE1 deletion in APCs, with implications in Th17 induction.[57] Future work should directly assess intestinal permeability and clarify whether pre-existing type 3 immunity poises the tissue towards inflammatory and autoimmune features upon challenge.

In addition, this study reveals that the IRE1 control over type 3 immunity via APCs does not require the transcription factor XBP1s, as XBP1$^{\Delta DC}$ mice do not replicate the phenotype observed in IRE1^{TruncDC} mice, suggesting that IRE1 RNase in APCs may restrain type 3 immunity through selective miRNA and mRNA degradation via RIDD. Along these lines, pre-miRNA preservation upon RIDD inhibition has been previously observed in hepatocytes.[89] Future studies should identify specific miRNA and mRNA substrates of IRE1 competent to fine-tune intestinal immunity via APCs. Along these lines, our findings provide a relevant functional insight that decouples IRE1 RNase functions from the well-known XBP1s-associated mechanisms reported for other intestinal cells. For instance, intestinal ILC3s require an intact IRE1 RNase–XBP1s pathway for optimal production of IL22,[60] and XBP1s deficiency in IECs

enhances Th17 responses through ROS generation and increased purine metabolite synthesis.[57] Our study shows that IRE1 RNase loss in CD11c$^+$ cells similarly augments type 3 immune responses, albeit independent of XBP1s presence, highlighting the multifaceted and cell-type–specific roles of the IRE1–XBP1s pathway and reinforcing its role as a key regulator of type 3 immunity.

Furthermore, we describe that IRE1 deficiency reprograms the profile of intestinal DCs toward a pro-type 3 profile. IRE1-deficient SI-LP cDCs increase IL6, IL1β, and

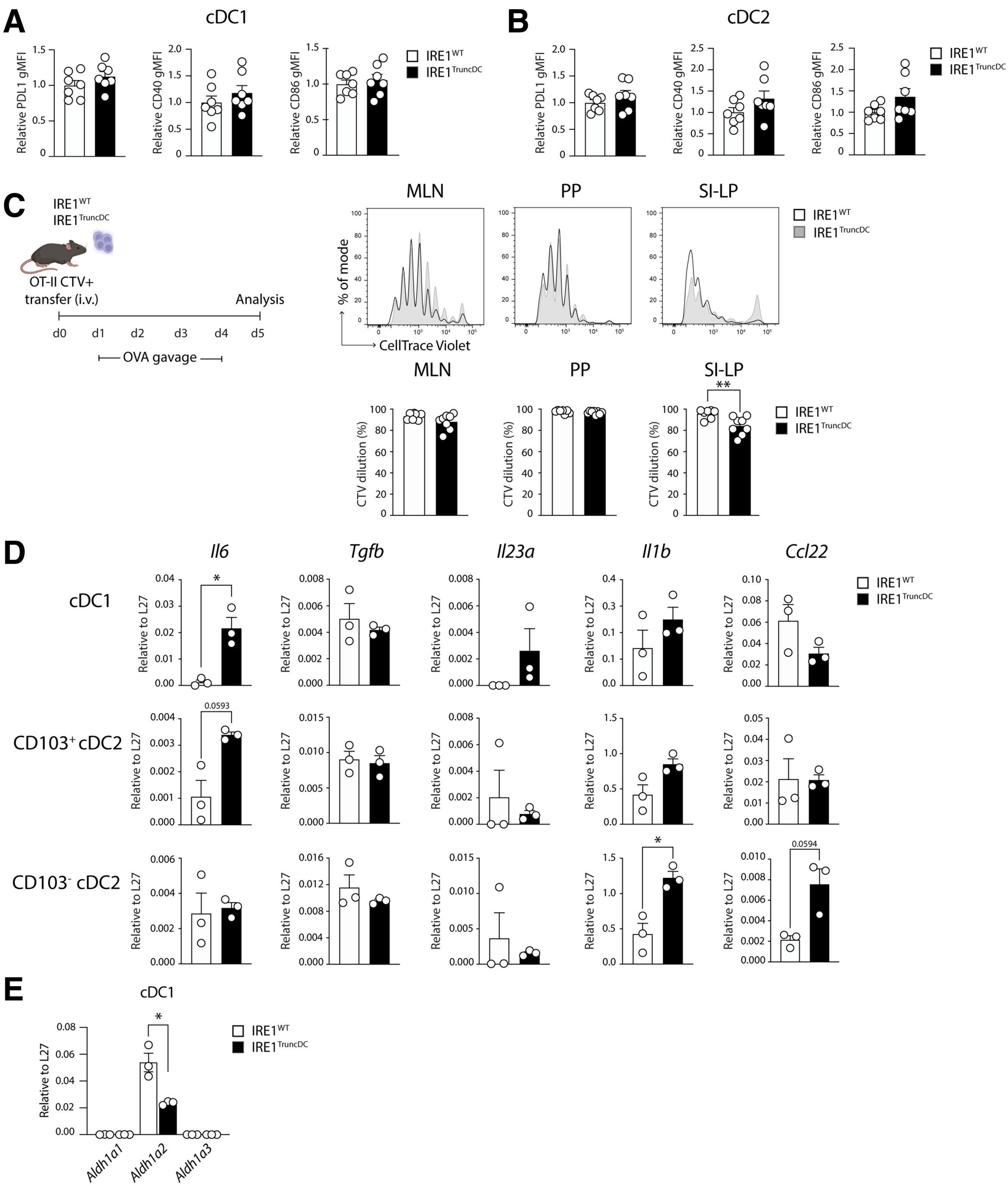

CCL22 transcription, all of which are known to be involved in the priming and recruitment of Th17 cells.[7,25,29] In addition, IRE1 RNase deficiency also led to a pronounced reduction of IL22BP expression by cDC subtypes and CIA-DCs. IL22BP is a well-described negative regulator of IL22 bioavailability and ILC3 functions,[81] reported to be produced by various subtypes of intestinal APCs.[4,79,90,91,94]Therefore, decreased IL22BP levels are expected to increase type 3 immune response in IRE1^{TruncDC} mice. In line with these findings, IRE1^{TruncDC} mice phenocopy several features of IL22BP knockout mice, such as the reduced expression of CD36 and the impaired lipid uptake.[4,92] Future studies manipulating the IL22–IL22BP in IRE1^{TruncDC} mice are required to establish the necessity or sufficiency for the lipid uptake defects.

Overall, our findings suggest that the tissue responses observed in IRE1^{TruncDC} mice arise from reprogramming of multiple APC subtypes, particularly cDCs and CIA-DCs. This is relevant considering that the identity of the APC driving gut Th17 induction has remained inconclusive. Intestinal Th17 cells persist upon deletion of cDC1s, cDC2s, or both, whereas CSF1R-dependent macrophages regulate intestinal Th17 numbers.[10] It is important to note that IRE1^{TruncDC} mice show decreased numbers of SI-LP and migratory cDC1s in the MLNs.[64] However, the effects observed here are unlikely to be driven only by decreased cDC1 frequencies as cDC1 loss impairs intestinal Th1 but not Th17 responses.[93] Furthermore, the unaltered Foxp3$^+$/RORγt$^+$ compartment in IRE1^{TruncDC} mice also indicates that RORγt$^+$ APCs are not main IRE1 targets under steady state conditions, underscoring a selective regulatory function of IRE1 RNase in intestinal APC subsets. However, as the CD11c–Cre mice line targets several APCs and small populations of other cell types such as NK cells,[94] future work using cDCs, CIA-DCs, and macrophage- and monocyte-restricted Cre lines will be required to ascribe cell-intrinsic causality.

IL17 and IL22 are elevated in samples of patients with ulcerative colitis, with ER stress emerging as a common denominator of intestinal inflammation.[95,96] However, the extent of IRE1 RNase activation, considering RIDD and XBP1s outputs, remain to be defined. Importantly, IL17A blockade with a monoclonal antibody (secukinumab) has not benefited patients with inflammatory bowel disease in clinical trials and showed unfavorable responses,[97] underscoring the complexity of type 3 immunity in intestinal pathology. Given that IL22 is reported to protect mice from inflammatory bowel disease,[98] precise modulation of IRE1 RNase activity in gut DCs may represent an interesting therapeutic avenue to enhance protective IL22 and IL17 responses in absence of pathologic inflammation, warranting further investigations.

To sum up, our study identifies IRE1 in CD11c$^+$ APCs as a central regulator of intestinal immune balance. These findings not only redefine the complex roles of the UPR in intestinal immunity, but also open new therapeutic avenues aimed at fine-tuning IRE1 signaling in APCs to control barrier protection and restore mucosal equilibrium.

Material and Methods

Study Approval

All animal procedures were approved by bioethical committees and performed in accordance with institutional guidelines for animal care of the Fundación Ciencia y Vida and Universidad San Sebastian and the Faculty of Medicine, University of Chile.

Mice

ERAI,[65] XBP1^{WT} (XBP1fl/fl[74]), XBP1$^{\Delta DC}$ (XBP1fl/fl x CD11c-Cre[69]), IRE1^{WT} (IRE1fl/fl[70]), IRE1^{TruncDC} (IRE1fl/fl x CD11c-Cre), and OT-II mice were bred at Universidad de Chile and Fundación Ciencia y Vida in specific pathogen-free conditions. All mice were kept on a C57BL/6 background. Litters with mice of both sexes at 8 to 14 weeks of age were used for experiments.

Antibiotic Treatment

WT and ERAI mice were administered an antibiotic cocktail for 10 consecutive days via oral gavage. Each treatment consisted of 200 μL of autoclaved water, either alone or supplemented with the following antibiotics: ampicillin (1 mg/mL), kanamycin (1 mg/mL), gentamicin (1 mg/mL), metronidazole (1 mg/mL), neomycin (1 mg/mL), and vancomycin (0.5 mg/mL). Then, mice were sacrificed, and samples from organs were collected for further analysis.

Figure 9. (***See previous page***). **IRE1 RNase deficiency alters cytokine profiles by intestinal DC subsets.** (*A*) Quantification of PD-L1, programmed death-ligand 1 (PD-L1), CD40, and CD86 expression (relative gMFI) in intestinal cDC1 cells from WT and IRE1^{TruncDC} mice. The relative gMFI has been calculated by normalizing absolute gMFI of cDC1 to the average gMFI of cDC1 from WT mice. n = 7 mice per group; data from 2 independent experiments; mean ± standard error of the mean (SEM). (*B*) Quantification of PD-L1, CD40, and CD86 expression (relative gMFI) in intestinal cDC2 cells from WT and IRE1-TruncDC mice. The relative gMFI has been calculated by normalizing absolute gMFI of cDC2 to the average gMFI of cDC2 from WT mice. n = 7 mice per group; data from 2 independent experiments; mean ± SEM. (*C*) Scheme of adoptive transfer experimental design. 2×10^6 CTV$^+$ OT-II cells were transferred into WT and IRE1^{TruncDC} mice, and OVA was gavaged at the indicated time points. Mice were analyzed at day 5. Representative histogram and quantification of cell proliferation dye on transferred cells in MLNs, spleen, and SI-LP of WT and IRE1^{TruncDC} mice. n = 7–8 mice per group; data from 3 independent experiments. (*D*) Relative expression of cytokines in intestinal DCs sorted from WT and IRE1^{TruncDC} mice determined by RT-PCR. n = 3 samples per group; each sample of sorted DCs comes from a pool of 4–5 mice; mean ± SEM. *$P < .05$. (*E*) Relative expression of *Aldh1a1*, *Aldh1a2*, and *Aldh1a3* in sorted cDC1 from SI-LP of WT and IRE1^{TruncDC} mice determined by RT-PCR. n = 3 samples per group; each sample of sorted DCs comes from a pool of 4–5 mice; mean ± SEM. *$P < .05$. Data in (*A–C*) were analyzed with the Mann–Whitney test. Data in (*D* and *E*) were analyzed with the unpaired *t* test with Welch's correction.

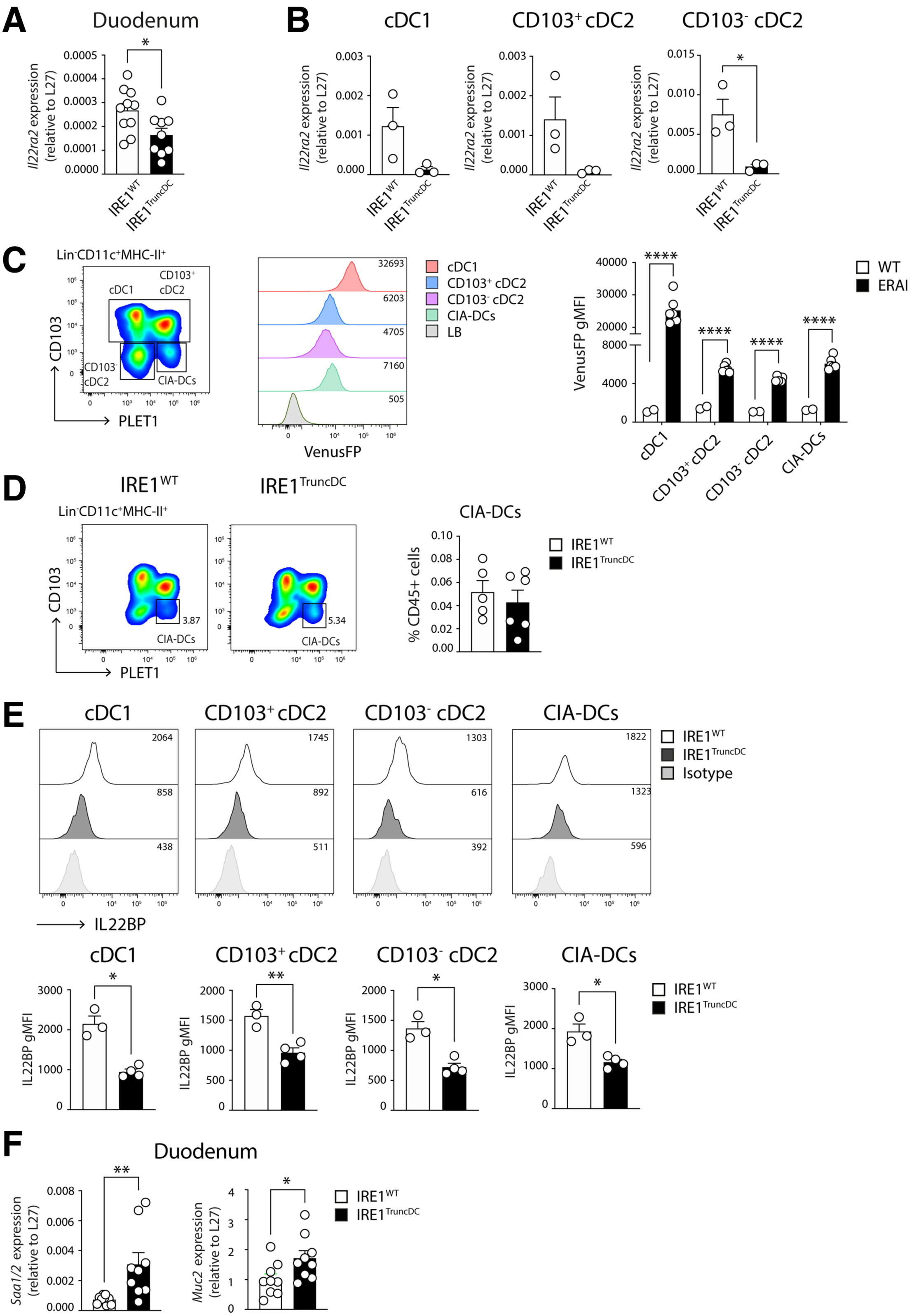
A
Duodenum
Il22ra2 expression (relative to L27)
IRE1WT
IRE1TruncDC
B
cDC1
CD103+ cDC2
CD103- cDC2
C
Lin-CD11c+MHC-II+
cDC1
CD103+ cDC2
CD103- cDC2
CIA-DCs
CD103
PLET1
32693
6203
4705
7160
505
VenusFP
cDC1
CD103+ cDC2
CD103- cDC2
CIA-DCs
LB
VenusFP gMFI
WT
ERAI
****
D
IRE1WT
IRE1TruncDC
Lin-CD11c+MHC-II+
3.87
5.34
CIA-DCs
% CD45+ cells
E
cDC1
CD103+ cDC2
CD103- cDC2
CIA-DCs
2064
858
438
1745
892
511
1303
616
392
1822
1323
596
IL22BP
IRE1WT
IRE1TruncDC
Isotype
IL22BP gMFI
F
Duodenum
Saa1/2 expression (relative to L27)
Muc2 expression (relative to L27)

Preparation of Cell Suspensions

Spleen and MLNs were minced and digested with collagenase D (1 mg/mL, Roche) and DNAse I (50 μg/mL, Roche) for 45 minutes at 37 °C in a water bath. Digested tissue was then passed through a 70-mm cell strainer, and single cells were kept on ice. For spleens, red blood cells were lysed using RBC lysis buffer (Biolegend).

Intestines were cleaned with Hank's balanced salt solution (HBSS), and fat and Peyer's patches were removed. Epithelial cells and intraepithelial lymphocytes were removed by incubating the tissues for 20 minutes at 37 °C with constant stirring at 100 rpm in serum-free medium (RPMI 1640 medium with 25 mM HEPES and 55 μM β-mercaptoethanol) supplemented with 0.94 mM dithiothreitol and 5 mM EDTA. The buffer was discarded, and the tissues were minced and incubated in serum-free medium supplemented with liberase TL (0.1 mg/mL, Roche) and DNAse I (0.5 μg/mL, Roche) for 30 minutes at 37 °C with constant stirring at 100 rpm. Digested tissue was then passed through a 100-μm cell strainer. Leukocytes were enriched by density gradient centrifugation with 40/75 Percoll (Cytiva) for 20 minutes at 600 × g.

Flow Cytometry and Cell Sorting

For surface staining, cells were incubated with anti-Fc receptor antibody and then stained with fluorochrome-conjugated antibodies (Supplementary Table 1) in fluorescence-activated cell sorting (FACS) buffer (phosphate-buffered saline [PBS] + 1% fetal bovine serum [FBS] + 2mM EDTA) for 30 minutes at 4 °C. Viability was assessed by staining with fixable viability Zombie (BioLegend) or LIVE/DEAD fixable (Invitrogen). Flow cytometry was performed on BD Fortessa LSR, BD FACS Verse, Cytek Northern Lights, or Cytek Aurora instruments. Analysis of flow cytometry data was done using FlowJo or Omiq software. Cell sorting was performed using a BD FACS Aria II.

Transcription Factors Intracellular Staining

After surface staining, cells were fixed and permeabilized using Foxp3 transcription factor staining set (eBioscience) followed by intracellular staining of transcription factors (Foxp3 and RORγt) as indicated by the manufacturer protocol.

T Cell Stimulation and Intracellular Cytokine Staining

SI-LP, MLN, and spleen cell suspensions were stimulated ex vivo prior to staining with 0.25 μM phorbol 12-myristate 13-acetate (Sigma) and 1 μg/mL ionomycin (Sigma) at 37 °C and 5% CO_2 for 3.5 hours in the presence of 1 μL/mL Brefeldin A (BD GolgiPlug). After stimulation, cells were surface stained as mentioned above. Then, cells were fixed and permeabilized using the BD Cytofix/Cytoperm fixation and permeabilization kit (BD Biosciences) followed by the intracellular staining of cytokines (IFN-γ, IL17, and IL22) as indicated by the manufacturer protocol.

Adoptive T Cell Transfer

$CD4^+$ T cells were purified from the spleens of OT-II mice. The spleen was perfused with RPMI + 10% FBS, and red blood cells were lysed using RBC lysis buffer (Biolegend). CD4+ T cells were isolated using the $CD4^+$ T Cell Isolation Kit (Miltenyi Biotec) following the manufacturer's instructions. Purified $CD4^+$ T cells were stained with CellTrace Violet (Invitrogen) at 5 μM. Labeled OT-II $CD4^+$ T cells were resuspended in PBS, and 2×10^6 $CD4^+$ T cells were intravenously transferred into WT and $IRE1^{TruncDC}$ mice.

Oral Ovalbumin Administration

OVA (A5503; Sigma-Aldrich) was administered orally using plastic gavage needles. Fifty milligrams in 200 μL PBS OVA was administered on days 1 and 2 after OT-II $CD4^+$ T cell transfer, and 25 mg in 100 μL PBS OVA on days 3 and 4 until the experiment end point on day 5.

Ex Vivo Assay for Lipid Absorption by Intestinal Epithelial Cells by Flow Cytometry

Lipid absorption by intestinal epithelial cells was performed as previously described.[4] Briefly, the small intestine was collected, and tissue was cleaned from feces and fat. The small intestine was sectioned into the proximal third (duodenum), middle third (jejunum), and distal third

Figure 10. (***See previous page***). **IRE1 RNase controls IL22BP by intestinal DC subtypes.** (*A*) Relative abundance of *Il22ra2* in the duodenum of WT and $IRE1^{TruncDC}$ mice determined by RT-PCR. n = 9–10 mice per group; data pooled from 3 independent experiments; mean ± standard error of the mean (SEM). **P* < .05. (*B*) Relative abundance of *Il22ra2* in DC subsets sorted from the SI-LP of WT and $IRE1^{TruncDC}$ mice determined by RT-PCR. n = 3 samples per group; each sample of sorted DCs comes from a pool of 4–5 mice; mean ± SEM. **P* < .05. (*C*) Representative flow cytometry plots of SI-LP DC subtypes (Lin(CD64, CX3CR1, B220, Ly6G)$^-$ $CD11c^+$ MHC-II$^+$). Representative histograms and quantification of VenusFP fluorescence (gMFI) within intestinal DCs from ERAI transgenic animals compared with WT mice. n = 2–6 mice per group; data pooled from 2 independent experiments; mean ± SEM. *****P* < .0001. (*D*) Representative flow cytometry plots (gate $CD11c^+$ MHC-II$^+$) and quantification of CIA-DCs within the $CD45^+$ compartment from the SI-LP of WT and $IRE1^{TruncDC}$ mice. n = 5–6 mice per group, data pooled from 3 independent experiments; mean ± SEM. (*E*) Representative histograms and quantification of IL22BP fluorescence (gMFI) within DCs from SI-LP of WT and $IRE1^{TruncDC}$ mice. n = 3–4 mice per group; mean ± SEM.**P* < .05, ***P* < .01. (*F*) Relative abundance of *Saa1/2* and *Muc2* in the duodenum of WT and $IRE1^{TruncDC}$ mice determined by RT-PCR. n = 9 mice per group; data pooled from 3 independent experiments; mean ± SEM. **P* < .05. Data in (*A*, *D* and *F*) were analyzed with the Mann–Whitney test. Data in (*B*, *C* and E) were analyzed with the unpaired *t* test with Welch's correction. MHC-II, major histocompatibility complex class II.

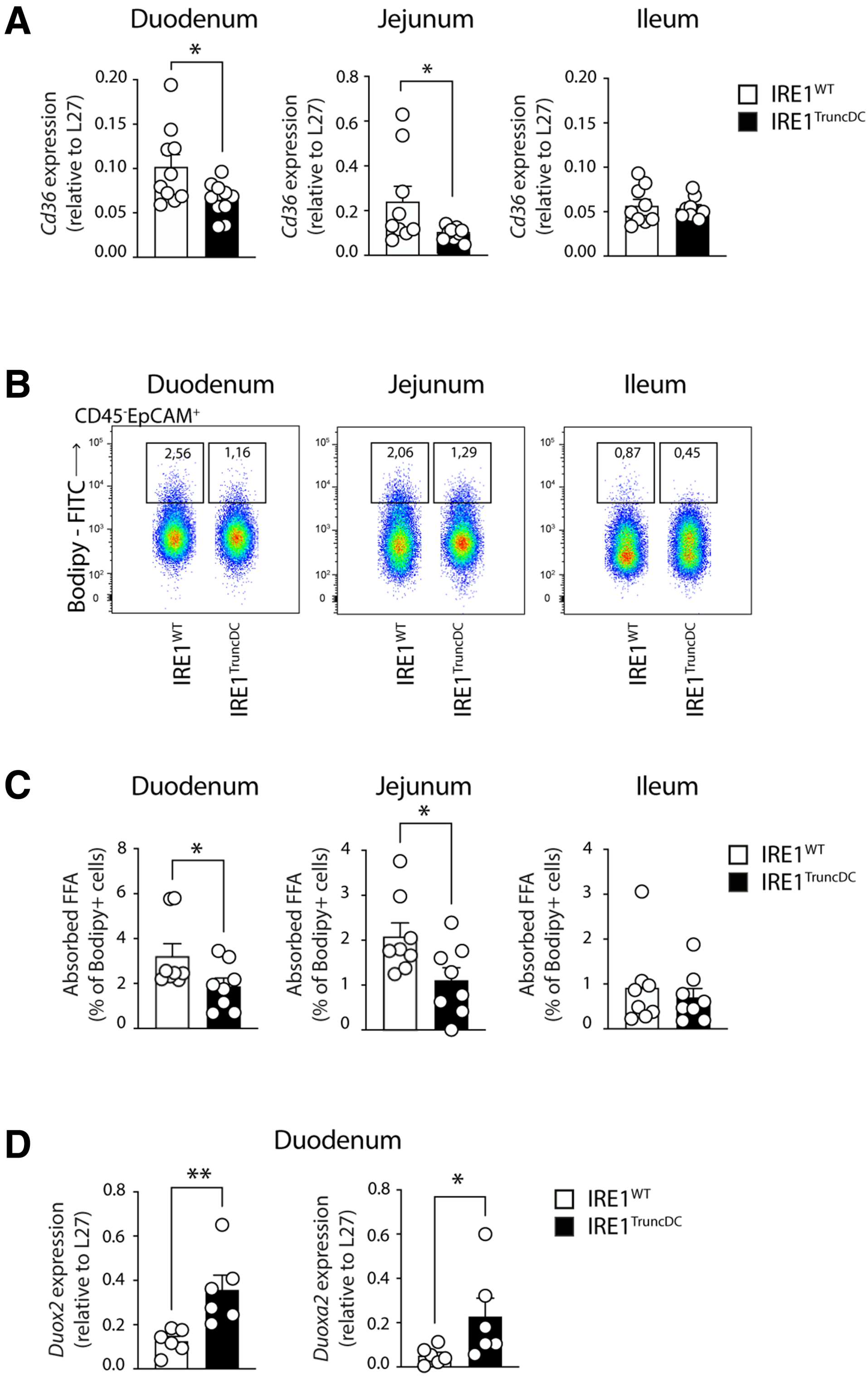

Figure 11. IRE1^{TruncDC} mice exhibit altered lipid uptake. (*A*) Relative abundance of *Cd36* in the duodenum, jejunum, and ileum of WT and IRE1^{TruncDC} mice determined by RT-PCR. n = 9 mice per group; data pooled from 3 independent experiments; mean ± standard error of the mean (SEM). *$P < .05$. (*B*) Representative histograms of lipid absorption of small intestinal epithelial cells from WT and IRE1^{TruncDC} mice. Epithelial cells were isolated from the indicated regions of the small intestine, and absorption of fluorescently labeled FAA (Bodipy FL C_{12}) was measured by flow cytometry. (*C*) Quantification of the percentage of epithelial cells that have taken up fluorescently labeled FFA. n = 8 mice per group; mean ± SEM. *$P < .05$. (*D*) Relative abundance of *Duox2* and *Duoxa2* in the duodenum of WT and IRE1^{TruncDC} mice determined by RT-PCR. n = 6 mice per group; data pooled from 2 independent experiments; mean ± SEM. *$P < .05$, **$P < .01$. All data were analyzed with the Mann–Whitney test.

(ileum). The epithelial layer was dissociated by incubating the tissue for 40 minutes in 10 mL HBSS supplemented with 5 mM EDTA and 10 mM HEPES at 37 °C with constant stirring at 100 rpm. Following this, mechanical isolation of the epithelial fraction was obtained by vigorous vortexing of the tissue for 40 seconds. The tissue was discarded, and the entire epithelial fraction was collected and washed twice with 10 mL cold PBS. The washed epithelial cells were then filtered using a 100-μm cell strainer. The epithelial cells were stained with CD45, CD306 (EPCAM), and LIVE/DEAD fixable (Invitrogen) for 20 minutes at 4 °C. Following staining, the cells were washed with PBS + 0.1% bovine serum albumin (BSA) and resuspended in 150 μL of warm HBSS + 0.1% fatty acid–free BSA + Bodipy FL C_{12} 2 μM (D3822, Invitrogen) and incubated in the water bath at 37 °C for 30 minutes. The lipid absorption assay was stopped using ice-cold PBS + 0.2% BSA. The cells were analyzed immediately.

RNA Isolation and Complementary DNA Generation

Total RNA was extracted from sorted cDCs from SI-LP with the RNeasy Micro Kit (Qiagen) and from murine intestinal tissue with Trizol reagent (Life Technologies) after the homogenization of the tissue using 3-mm zirconium beads and the BeadBug homogenizer. RNA from the duodenum was reverse-transcribed with a mixture of Random Hexamer Primer (Thermofisher) and with the M-MLV reverse transcriptase Kit (Invitrogen). Complementary DNA from sorted cells was synthesized and amplified using the Ovation PicoSL WTA System V2 (Nugen).

Quantitative Polymerase Chain Reaction

qPCR was performed with a SYBR Green PCR Master Mix kit (Applied Biosystems). Quantification of the PCR signals of each sample was performed by comparing the cycle threshold (Ct) values, in duplicate, of the gene of interest with the Ct values of the L27 housekeeping gene. See Supplementary Table 2 for primers used for qPCR.

Cytokine Secretion Measurement

Tissue sections 1 cm in length of the different intestinal segments were placed into RPMI medium (Corning) containing 2% FBS and incubated for 24 hours at 37 °C with 5% CO_2. The supernatants were collected and stored before analysis at −80 °C. The supernatants were analyzed using the CBA Kit Mouse Th1/Th2/Th17 (BD Biosciences) following the manufacturer's instructions.

Histology

Formalin-fixed, paraffin-embedded sections of the small intestine were stained with hematoxylin and eosin or Periodic acid-Schiff. The number of goblet cells (Periodic acid-Schiff+ cells along the villus length) was determined under light microscopy; 10 villi from at least 2 sections per animal were evaluated.

16S Ribosomal RNA Gene Quantitative Polymerase Chain Reaction Analysis

Fecal pellets were collected from IRE1^{TruncDC} and control littermates and frozen at −80 °C. Fecal material was subjected to the QIAmp Fast DNA Stool isolation kit (Qiagen) following the provider's instructions. Total DNA was quantified using Nanodrop One C (Thermo Fisher), and 10 ng/mL was used to set up the qPCR reaction. The primers for the bacterial genes are indicated in.[99] For analysis, relative expression was determined using the $\Delta\Delta$Ct method, using 16S as a housekeeping control. To calculate the relative abundance, we considered the sum of all bacteria genera tested as total microbiota or 100% and then obtained the relative presence for each one. Segmented filamentous bacteria primers were also included in this analysis.[100]

Statistical Analysis

Statistical analysis was conducted using GraphPad Prism software (v9.1.2). Results are presented as the mean ± standard error of the mean. To ensure a normal distribution, data were subjected to the Shapiro–Wilk test. Two groups were compared using the 2-tailed *t* test with Welch's correction (if the 2 populations do not have the same standard deviation) or using a nonparametric 2-tailed Mann–Whitney test as indicated in figure legends. Multiple groups were compared using 1-way analysis of variance with Šídák's post-test or using the nonparametric Kruskal–Wallis test as indicated in figure legends. A P value $< .05$ was considered statistically significant.

Supplementary Material

Note: To access the supplementary material accompanying this article, visit the full text version at https://doi.org/10.1016/j.jcmgh.2026.101877.

References

1. Sun T, Nguyen A, Gommerman JL. Dendritic cell subsets in intestinal immunity and inflammation. J Immunol 2020;204:1075–1083.
2. Mora JR, Bono MR, Manjunath N, et al. Selective imprinting of gut-homing T cells by Peyer's Patch dendritic cells. Nature 2003;424:88–93.
3. Esterházy D, Canesso MCC, Mesin L, et al. Compartmentalized gut lymph node drainage dictates adaptive immune responses. Nature 2019;569:126–130.
4. Guendel F, Kofoed-Branzk M, Gronke K, et al. Group 3 innate lymphoid cells program a distinct subset of IL-22BP-producing dendritic cells demarcating solitary intestinal lymphoid tissues. Immunity 2020; 53:1015–1032.e8.
5. Scott CL, Bain CC, Wright PB, et al. CCR2+CD103− intestinal dendritic cells develop from DC-committed precursors and induce interleukin-17 production by T cells. Mucosal Immunol 2015;8:327–339.
6. Bogunovic M, Ginhoux F, Helft J, et al. Origin of the lamina propria dendritic cell network. Immunity 2009; 31:513–525.

7. Persson EK, Uronen-Hansson H, Semmrich M, et al. IRF4 transcription-factor-dependent CD103+CD11b+ dendritic cells drive mucosal T helper 17 cell differentiation. Immunity 2013;38:958–969.
8. Merad M, Sathe P, Helft J, et al. The dendritic cell lineage: ontogeny and function of dendritic cells and their subsets in the steady state and the inflamed setting. Annu Rev Immunol 2013;31:563–604.
9. Cabeza-Cabrerizo M, Cardoso A, Minutti CM, et al. Dendritic cells revisited. Annu Rev Immunol 2021;39:131–166.
10. Panea C, Farkas AM, Goto Y, et al. Intestinal monocyte-derived macrophages control commensal-specific Th17 responses. Cell Rep 2015;12:1314–1324.
11. Rodrigues PF, Wu S, Trsan T, et al. Rorγt-positive dendritic cells are required for the induction of peripheral regulatory T cells in response to oral antigens. Cell 2025;188:2720–2737.e22.
12. Cabric V, Franco Parisotto Y, Park T, et al. A wave of Thetis cells imparts tolerance to food antigens early in life. Science 2025;389:268–274.
13. Fu L, Upadhyay R, Pokrovskii M, et al. PRDM16-dependent antigen-presenting cells induce tolerance to gut antigens. Nature 2025;642:756–765.
14. Kedmi R, Najar TA, Mesa KR, et al. A RORγt+ cell instructs gut microbiota-specific Treg cell differentiation. Nature 2022;610:737–743.
15. Akagbosu B, Tayyebi Z, Shibu G, et al. Novel antigen-presenting cell imparts Treg-dependent tolerance to gut microbiota. Nature 2022;610:752–760.
16. Sun IH, Qualls AE, Yin HS, et al. RORγt eTACs mediate oral tolerance and Treg induction. J Exp Med 2025;222.
17. Canesso MCC, de Castro TBR, Nakandakari-Higa S, et al. Identification of antigen-presenting cell–T cell interactions driving immune responses to food. Science 2025;387:eado5088.
18. Zhou W, Zhou JZ, Ahmed A, et al. ILC3s sense gut microbiota through STING to initiate immune tolerance. Immunity 2025;58:1762–1777.
19. Narasimhan H, Richter ML, Shakiba R, et al. RORγt-expressing dendritic cells are functionally versatile and evolutionarily conserved antigen-presenting cells. Proc Natl Acad Sci U S A 2025;122:e2417308122.
20. Schnell A, Littman DR, Kuchroo VK. TH17 cell heterogeneity and its role in tissue inflammation. Nat Immunol 2023;24:19–29.
21. Ryu S, Lim MY, Kim J, et al. Versatile roles of innate lymphoid cells at the mucosal barrier: from homeostasis to pathological inflammation. Exp Mol Med 2023;55:1845–1857.
22. Mills KHG. IL-17 and IL-17-producing cells in protection versus pathology. Nat Rev Immunol 2022;23:38–54.
23. Annunziato F, Romagnani C, Romagnani S. The 3 major types of innate and adaptive cell-mediated effector immunity. J Allergy Clin Immunol 2015;135:626–635.
24. Fabre T, Molina MF, Soucy G, et al. Type 3 cytokines IL-17A and IL-22 drive TGF-β–dependent liver fibrosis. Sci Immunol 2018;3:7754.
25. Schlitzer A, McGovern N, Teo P, et al. IRF4 transcription factor-dependent CD11b+ dendritic cells in human and mouse control mucosal IL-17 cytokine responses. Immunity 2013;38:970–983.
26. Longman RS, Diehl GE, Victorio DA, et al. CX_3CR1^+ mononuclear phagocytes support colitis-associated innate lymphoid cell production of IL-22. J Exp Med 2014;211:1571–1583.
27. Dobeš J, Ben-Nun O, Binyamin A, et al. Extrathymic expression of Aire controls the induction of effective TH17 cell-mediated immune response to Candida Albicans. Nat Immunol 2022;23:1098–1108.
28. Liang J, Huang HI, Benzatti FP, et al. Inflammatory Th1 and Th17 in the Intestine Are Each Driven by Functionally Specialized Dendritic Cells with Distinct Requirements for MyD88. Cell Rep 2016;17:1330–1343.
29. Kinnebrew MA, Buffie CG, Diehl GE, et al. Interleukin 23 production by intestinal CD103(+)CD11b(+) dendritic cells in response to bacterial flagellin enhances mucosal innate immune defense. Immunity 2012;36:276–287.
30. Sano T, Kageyama T, Fang V, et al. Redundant cytokine requirement for intestinal microbiota-induced Th17 cell differentiation in draining lymph nodes. Cell Rep 2021;36:109608.
31. Jie Z, Yang JY, Gu M, et al. NIK signaling axis regulates dendritic cell function in intestinal immunity and homeostasis. Nat Immunol 2018;19:1224–1235.
32. Coleman OI, Haller D. ER stress and the UPR in shaping intestinal tissue homeostasis and immunity. Front Immunol 2019;10:2825.
33. Bettigole SE, Glimcher LH. Endoplasmic reticulum stress in immunity. Annu Rev Immunol 2015;33:107–138.
34. Oakes SA, Papa FR. The role of endoplasmic reticulum stress in human pathology. Annu Rev Pathol 2014;10:173–194.
35. Grootjans J, Kaser A, Kaufman RJ, et al. The unfolded protein response in immunity and inflammation. Nat Rev Immunol 2016;16:469–484.
36. Conza GD, Ho P-C, Cubillos-Ruiz JR, et al. Control of immune cell function by the unfolded protein response. Nat Rev Immunol 2023;23:546–562.
37. Janssens S, Pulendran B, Lambrecht BN. Emerging functions of the unfolded protein response in immunity. Nat Immunol 2014;15:910–919.
38. Flores-Santibáñez F, Medel B, Bernales JI, et al. Understanding the role of the unfolded protein response sensor IRE1 in the biology of antigen presenting cells. Cells 2019;8:1563.
39. Mogilenko DA, Haas JT, L'homme L, et al. Metabolic and innate immune cues merge into a specific inflammatory response via the UPR. Cell 2019;177:1201–1216.e19.
40. Urano F, Wang X, Bertolotti A, et al. Coupling of stress in the ER to activation of JNK protein kinases by transmembrane protein kinase IRE1. Science 2000;287:664–666.
41. Hu P, Han Z, Couvillon AD, et al. Autocrine tumor necrosis factor alpha links endoplasmic reticulum stress to the membrane death receptor pathway through IRE1alpha-mediated NF-kappaB activation and down-regulation of TRAF2 expression. Mol Cell Biol 2006;26:3071–3084.

42. Keestra-Gounder AM, Byndloss MX, Seyffert N, et al. NOD1 and NOD2 signalling links ER stress with inflammation. Nature 2016;532:394–397.
43. Gendrisch F, Völkel L, Fluck M, et al. IRE1 and PERK signaling regulates inflammatory responses in a murine model of contact hypersensitivity. Allergy 2022; 77:966–978.
44. Walter P, Ron D. The unfolded protein response: From stress pathway to homeostatic regulation. Science 2011;334:1081–1086.
45. Yoshida H, Matsui T, Yamamoto A, et al. XBP1 mRNA is induced by ATF6 and spliced by IRE1 in response to ER stress to produce a highly active transcription factor. Cell 2001;107:881–891.
46. Calfon M, Zeng H, Urano F, et al. IRE1 couples endoplasmic reticulum load to secretory capacity by processing the XBP-1 mRNA. Nature 2002;415:92–96.
47. Hollien J, Lin JH, Li H, et al. Regulated Ire1-dependent decay of messenger RNAs in mammalian cells. J Cell Biol 2009;186:323–331.
48. Maurel M, Chevet E, Tavernier J, et al. Getting RIDD of RNA: IRE1 in cell fate regulation. Trends Biochem Sci 2014;39:245–254.
49. So JS, Hur KY, Tarrio M, et al. Silencing of lipid metabolism genes through ire1α-mediated Mrna decay lowers plasma lipids in mice. Cell Metab 2012; 16:487–499.
50. Tang C-HA, Chang S, Paton AW, et al. Phosphorylation of IRE1 at S729 regulates RIDD in B cells and antibody production after immunization. J Cell Biol 2018; 217:1739–1755.
51. Shan B, Wang X, Wu Y, et al. The metabolic ER stress sensor IRE1α suppresses alternative activation of macrophages and impairs energy expenditure in obesity. Nat Immunol 2017;18:519–529.
52. Almanza A, Mnich K, Blomme A, et al. Regulated IRE1α-dependent decay (RIDD)-mediated reprograming of lipid metabolism in cancer. Nat Commun 2022;13:2493.
53. Lira-Barraza A, López-Schettini J, Osorio F. Immunoregulatory functions of the endoplasmic reticulum stress sensor inositol-requiring enzyme 1 in myeloid cell biology. J Leukoc Biol 2025;117.
54. Welz L, Kakavand N, Hang X, et al. Epithelial X-box binding protein 1 coordinates tumor protein p53-driven DNA damage responses and suppression of intestinal carcinogenesis. Gastroenterology 2022;162:223–237.e11.
55. Niederreiter L, Fritz TM, Adolph TE, et al. ER stress transcription factor Xbp1 suppresses intestinal tumorigenesis and directs intestinal stem cells. J Exp Med 2013;210:2041–2056.
56. Grootjans J, Krupka N, Hosomi S, et al. Epithelial endoplasmic reticulum stress orchestrates a protective IgA response. Science 2019;363:993–998.
57. Duan J, Matute JD, Unger LW, et al. Endoplasmic reticulum stress in the intestinal epithelium initiates purine metabolite synthesis and promotes Th17 cell differentiation in the gut. Immunity 2023;56:1115–1131.e9.
58. Adolph TE, Tomczak MF, Niederreiter L, et al. Paneth cells as a site of origin for intestinal inflammation. Nature 2013;503:272–276.
59. Tschurtschenthaler M, Adolph TE, Ashcroft JW, et al. Defective ATG16L1-mediated removal of IRE1α drives Crohn's disease-like ileitis. J Exp Med 2017; 214:401–422.
60. Cao S, Fachi JL, Ma K, et al. The IRE1α/XBP1 pathway sustains cytokine responses of group 3 innate lymphoid cells in inflammatory bowel disease. J Clin Invest 2024; 134.
61. Kaser A, Lee AH, Franke A, et al. XBP1 links ER stress to intestinal inflammation and confers genetic risk for human inflammatory bowel disease. Cell 2008; 134:743–756.
62. Iwakoshi NN, Pypaert M, Glimcher LH. The transcription factor XBP-1 is essential for the development and survival of dendritic cells. J Exp Med 2007;204:2267–2275.
63. Osorio F, Tavernier SJ, Hoffmann E, et al. The unfolded-protein-response sensor IRE-1α regulates the function of CD8α+ dendritic cells. Nat Immunol 2014;15:248–257.
64. Tavernier SJJ, Osorio F, Vandersarren L, et al. Regulated IRE1-dependent mRNA decay sets the threshold for dendritic cell survival. Nat Cell Biol 2017;19:698–710.
65. Iwawaki T, Akai R, Kohno K, et al. A transgenic mouse model for monitoring endoplasmic reticulum stress. Nat Med 2004;10:98–102.
66. Flores-Santibañez F, Rennen S, Fernández D, et al. Nuanced role for dendritic cell intrinsic IRE1 RNase in the regulation of antitumor adaptive immunity. Front Immunol 2023;14:1–14.
67. Guilliams M, Dutertre CA, Scott CL, et al. Unsupervised high-dimensional analysis aligns dendritic cells across tissues and species. Immunity 2016;45:669–684.
68. Kaser A, Blumberg RS. Introduction: the unfolded protein response's role in disease pathophysiology. Semin Immunopathol 2013;35:255–257.
69. Caton ML, Smith-Raska MR, Reizis B. Notch-RBP-J signaling controls the homeostasis of CD8- dendritic cells in the spleen. J Exp Med 2007;204:1653–1664.
70. Iwawaki T, Akai R, Yamanaka S, et al. Function of IRE1 alpha in the placenta is essential for placental development and embryonic viability. Proc Natl Acad Sci U S A 2009;106:16657–16662.
71. Arnold IC, Mathisen S, Schulthess J, et al. CD11c+ monocyte/macrophages promote chronic Helicobacter hepaticus-induced intestinal inflammation through the production of IL-23. Mucosal Immunol 2016;9:352–363.
72. Luda KM, Joeris T, Persson EK, et al. IRF8 transcription-factor-dependent classical dendritic cells are essential for intestinal t cell homeostasis. Immunity 2016; 44:860–874.
73. Schnell A, Huang L, Singer M, et al. Stem-like intestinal Th17 cells give rise to pathogenic effector T cells during autoimmunity. Cell 2021;184:6281–6298.e23.
74. Lee A, Scapa E, Cohen D, et al. Regulation of hepatic lipogenesis by the transcription factor XBP1. Science 2008;320:1492–1496.
75. Miossec P, Kolls JK. Targeting IL-17 and TH17 cells in chronic inflammation. Nat Rev Drug Discov 2012; 11:763–776.
76. Wang A, Wang Z, Cao Y, et al. CCL2/CCR2-dependent recruitment of Th17 cells but not Tc17 cells to the lung in

a murine asthma model. Int Arch Allergy Immunol 2015;166:52–62.
77. Sun CM, Hall JA, Blank RB, et al. Small intestine lamina propria dendritic cells promote de novo generation of Foxp3 T reg cells via retinoic acid. J Exp Med 2007;204:1775–1785.
78. Martin JC, Bériou G, Heslan M, et al. Interleukin-22 binding protein (IL-22BP) is constitutively expressed by a subset of conventional dendritic cells and is strongly induced by retinoic acid. Mucosal Immunol 2014;7:101–113.
79. Huber S, Gagliani N, Zenewicz LA, et al. IL-22BP is regulated by the inflammasome and modulates tumorigenesis in the intestine. Nature 2012;491:259–263.
80. Depreter MGL, Blair NF, Gaskell TL, et al. Identification of Plet-1 as a specific marker of early thymic epithelial progenitor cells. Proc Natl Acad Sci U S A 2008;105:961–966.
81. Zenewicz LA. IL-22 binding protein (IL-22BP) in the regulation of IL-22 biology. Front Immunol 2021;12:1–10.
82. Sano T, Huang W, Hall JA, et al. An IL-23R/IL-22 circuit regulates epithelial serum amyloid A to promote local effector Th17 responses. Cell 2015;163:381–393.
83. Gaudino SJ, Beaupre M, Lin X, et al. IL-22 receptor signaling in Paneth cells is critical for their maturation, microbiota colonization, Th17-related immune responses, and anti-Salmonella immunity. Mucosal Immunol 2021;14:389–401.
84. Tiwari S, Begum S, Moreau F, et al. Autophagy is required during high MUC2 mucin biosynthesis in colonic goblet cells to contend metabolic stress. Am J Physiol Gastrointest Liver Physiol 2021;321:G489–G499.
85. Turner JE, Stockinger B, Helmby H. IL-22 mediates goblet cell hyperplasia and worm expulsion in intestinal helminth infection. PLoS Pathog 2013;9:e1003698.
86. Mao K, Baptista AP, Tamoutounour S, et al. Innate and adaptive lymphocytes sequentially shape the gut microbiota and lipid metabolism. Nature 2018;554:255–259.
87. Kaya GG, Schwarzer R, Dannappel M, et al. Unfolded protein response transcription factor XBP1 suppresses necroptosis-induced colitis by reinforcing the mucus barrier. Immunity 2025;58:2208–2225.e6.
88. Luo K, Cao SS. Endoplasmic reticulum stress in intestinal epithelial cell function and inflammatory bowel disease. Gastroenterol Res Pract 2015;2015:328791.
89. Wang J-M, Qiu Y, Yang Z, et al. IRE1α prevents hepatic steatosis by processing and promoting the degradation of select microRNAs. Sci Signal 2018;11.
90. Silva CD, Wagner C, Bonnardel J, et al. The Peyer's patch mononuclear phagocyte system at steady state and during infection. Front Immunol 2017;8.
91. Jinnohara T, Kanaya T, Hase K, et al. IL-22BP dictates characteristics of Peyer's patch follicleassociated epithelium for antigen uptake. J Exp Med 2017;214:1607–1618.
92. Laaksonen H, Guerreiro-Cacais AO, Adzemovic MZ, et al. The multiple sclerosis risk gene IL22RA2 contributes to a more severe murine autoimmune neuroinflammation. Genes Immun 2014;15:457–465.
93. Ahmadi F, Junghus F, Ashworth C, et al. cDC1-derived IL-27 regulates small intestinal CD4+ T cell homeostasis in mice. J Exp Med 2023;220.
94. Abram CL, Roberge GL, Hu Y, et al. Comparative analysis of the efficiency and specificity of myeloid-Cre deleting strains using ROSA-EYFP reporter mice. J Immunol Methods 2014;408:89–100.
95. Rodrigues BL, Dotti I, Pascoal LB, et al. Endoplasmic reticulum stress in colonic mucosa of ulcerative colitis patients is mediated by PERK and IRE1 pathway activation. Mediators Inflamm 2022;2022:6049500.
96. Kaser A, Blumberg RS. Endoplasmic reticulum stress and intestinal inflammation. Mucosal Immunol 2010;3:11–16.
97. Hueber W, Sands BE, Lewitzky S, et al. Secukinumab, a human anti-IL-17A monoclonal antibody, for moderate to severe Crohn's disease: unexpected results of a randomised, double-blind placebo-controlled trial. Gut 2012;61:1693–1700.
98. Zenewicz LA, Yancopoulos GD, Valenzuela DM, et al. Innate and adaptive interleukin-22 protects mice from inflammatory bowel disease. Immunity 2008;29:947–957.
99. Pinto C, Carrasco-Loncharic T, González-Mienert E, et al. IL-33 induces a switch in intestinal metabolites revealing the tryptophan pathway as a target for inducing allograft survival. Nutrients 2024;16:3655.
100. Barman M, Unold D, Shifley K, et al. Enteric salmonellosis disrupts the microbial ecology of the murine gastrointestinal tract. Infect Immun 2008;76:907–915.

Received November 24, 2025. Accepted August 27, 2026.

Correspondence
Address correspondence to: Fabiola Osorio, PhD, Laboratorio de Inmunología, Departamento de Biología, Facultad de Ciencias, Universidad de Chile, Las Palmeras 3425, Santiago 7800003, Chile. e-mail: fabiola.osorio@uchile.cl.

Acknowledgments
The authors thank the facilities at Universidad de Chile and Fundación Ciencia and Vida. The authors also thank the members of the immunology and cellular stress and immunology laboratories for critical support.

CRediT Authorship Contributions

Dominique Fernández, MSc (Conceptualization: Lead; Data curation: Lead; Formal analysis: Lead; Investigation: Lead; Methodology: Equal; Validation: Lead; Visualization: Equal; Writing – original draft: Supporting; Writing – review & editing: Supporting)

Antonia Geisse, MSc (Conceptualization: Equal; Data curation: Equal; Formal analysis: Equal; Investigation: Equal; Methodology: Equal; Visualization: Supporting; Writing – review & editing: Supporting)

Pamela González-Maldonado (Investigation: Supporting; Writing – review & editing: Supporting)

Ailiñ Tapia (Formal analysis: Supporting; Investigation: Supporting; Writing – review & editing: Supporting)

Francisca Gutiérrez-Ballesteros (Formal analysis: Lead; Investigation: Equal; Writing – review & editing: Supporting)

Valentina Ugalde (Investigation: Equal; Writing – review & editing: Supporting)

Felipe Tapia (Investigation: Supporting; Writing – review & editing: Supporting)

Felipe Flores-Santibañez, PhD (Investigation: Supporting; Writing – review & editing: Supporting)

Takao Iwawaki (Resources: Supporting; Writing – review & editing: Supporting)

Karina Pino-Lagos (Investigation: Supporting; Writing – review & editing: Supporting)
Louis Boon (Resources: Supporting; Writing – review & editing: Supporting)
Maria Rosa Bono (Resources: Supporting; Writing – review & editing: Supporting)
Carolina Prado (Methodology: Supporting; Resources: Supporting; Writing – review & editing: Supporting)
Rodrigo Pacheco (Methodology: Supporting; Resources: Supporting; Writing – review & editing: Supporting)
Fabiola Osorio, PhD (Conceptualization: Lead; Data curation: Supporting; Formal analysis: Equal; Funding acquisition: Lead; Investigation: Supporting; Methodology: Equal; Project administration: Lead; Resources: Lead; Supervision: Equal; Validation: Equal; Writing – original draft: Lead; Writing – review & editing: Lead)

Conflicts of interest

The authors disclose no conflicts.

Funding

This work was funded by a Fondo Nacional de Desarrollo Científico y Tecnológico (FONDECYT) regular grant No 1240337 (Fabiola Osorio) and 1230183 (Maria Rosa Bono); International Centre For Genetic Engineering and Biotechnology (ICGEB) Collaborative Research Grant CRP/CHL24-04 (Fabiola Osorio); Conicyt/Fondequip/EQM220027; Conicyt/Fondequip EQM140032; and by Centro Científico y Tecnológico de Excelencia Ciencia and Vida ANID-BASAL FB210008 (to Fabiola Osorio, Maria Rosa Bono, Carolina Prado, and Rodrigo Pacheco). MED.UCHILE-FACS Laboratory is supported by ANID through grants FONDEQUIP140032 (BD LSR Fortessa X-20, Special Order) and by the Biomedical Sciences Institute (ICBM), School of Medicine of University of Chile, Santiago de Chile.

Data Availability

The datasets generated during and/or analyzed during the current study are available from the corresponding author upon reasonable request.

Cellular and Molecular Gastroenterology and Hepatology, Volume *20*

Supplemental information

Control of Intestinal Type 3 Immunity by the Unfolded Protein Response Sensor Inositol-Requiring Enzyme 1α in Antigen-Presenting Cells

Dominique Fernández, Antonia Geisse, Pamela González-Maldonado, Ailiñ Tapia, Francisca Gutiérrez-Ballesteros, Valentina Ugalde, Felipe Tapia, Felipe Flores-Santibañez, Takao Iwawaki, Karina Pino-Lagos, Louis Boon, María Rosa Bono, Carolina Prado, Rodrigo Pacheco, and Fabiola Osorio

Supplementary Table I. Antibodies for flow cytometry and cell sorting

Antigen	Fluorophore	Company	Clone	Catalog
XCR1	PE	Biolegend	ZET	148204
CD3	PE-Cy5	Biolegend	145-2C11	100309
CD3	Brillian Violet 510	Biolegend	145-2C11	100353
B220	PE-Cy5	Biolegend	RA3-6B2	103209
B220	Brilliant Violet 510	Biolegend	RA3-6B2	103222
B220	Brilliant Violet 750	Biolegend	RA3-6B2	103239
CD172a	PE-Cy7	Biolegend	P84	144007
CD11c	APC	eBioscience	N418	17-0114-82
CD11c	Brilliant Violet 570	Biolegend	N418	117331
CD11c	PE	Biolegend	N418	117307
CD11c	PE-Cy7	eBioscience	N418	25-0114-82
CD11c	Brilliant Violet 711	Biolegend	N418	117349
Ly6G	Alexa Fluor 700	Biolegend	1A8	127622
MHC II	APC-Cy7	Biolegend	M5/114.15.2	107628
EpCAM	Brilliant Violet 421	Biolegend	G8.8	118225
Ly6C	Brillian Violet 605	Biolegend	HK1.4	128035
NK1.1	Brillian Violet 650	Biolegend	PK136	108736
CD64	Brillian Violet 711	Biolegend	X54-5/7.1	139311
CD64	PerCP-Cy5.5	Biolegend	X54-5/7.1	139307
CD64	PE Dazzle 594	Biolegend	X54-5/7.1	139319
CD11b	APC	Biolegend	M1/70	101211
CD11b	Brilliant Violet 786	Biolegend	M1/70	101243
CD45	PerCPCy5.5	Biolegend	30-F11	103132
CD45	Brillian UltraViolet 395	BD Biosciences	30-F11	564279
CD45	Brillian Violet 480	Invitrogen	30-F11	414-0451-80
CD45	Brilliant Violet 650	Biolegend	30-F11	103151
CD45.2	Alexa Fluor 647	Biolegend	104	109818
F4/80	Biotin	Biolegend	BM8	123105
CD103	PE-Cy7	Biolegend	2E7	121425
CD103	PerCP-eFluor 710	Invitrogen	2E7	121425
CD8	eFluor 450	eBioscience	53-6.7	48-0081-80
CX3CR1	Brilliant Violet 510	Biolegend	SA011F11	149015
CD4	PE Dazzle 594	Biolegend	GK1.5	100455

CD4	APC-Cy7	Tonbo Bioscience	GK1.5	25-0041
KLRG1	PerCP-Cy5.5	Biolegend	2F1/KLGR1	138418
CD127	PE-Cy7	Biolegend	A7R34	135014
CD90.2	APC-Cy7	Biolegend	30-H12	105328
CD90.2	FITC	Biolegend	30-H12	105305
TCRγδ	PerCPCy5.5	Biolegend	GL3	118117
IFNg	PE	eBioscience	XMG1.2	12-7311-82
IL-17	eFluor 660	Invitrogen	eBio17B7	50-7177-82
IL-17	PE-Cy7	Invitrogen	eBio17B7	25-7177-80
IL-17	BV605	BD Biosciences	TC11-18H10	564169
IL-22	PE	Biolegend	Poly5164	516404
RORγt	APC	Invitrogen	AFKJS-9	17-6988-80
RORγt	RB705	BD Biosciences	Q31-378	570259
FOXP3	PE-Cy7	Invitrogen	FJK-16s	25-5773-82
CXCR6	PE	Biolegend	SA051D1	151103
SLAMF6	APC	Biolegend	330-AJ	134609
SLAMF6	Pacific Blue	Biolegend	330-AJ	134608
PDL1	PE	Invitrogen	MIH5	12-5982-82
CD40	APC	Biolegend	3/23.	124611
CD86	PE-Cy7	Biolegend	GL-1	105013
Plet1	Purified	Depreter et al.[81]	1D4	
Goat anti-rat IgG	PE	Biolegend	Poly4054	405416
Goat anti-rat IgG	Alexa Fluor 647	Biolegend	Poly4054	405406
IL-22BP	APC	R&D Systems	Polyclonal	IC2376A
Isotype Sheep IgG (isotype IL-22BP)	APC	R&D Systems	Polyclonal	IC016A
CD16/32 (FcBlock)	Purified	Biolegend	93	101302
Streptavidin	PE-Cy7	Biolegend		405206
Streptavidin	BD Horizon BUV737	BD Biosciences		564293

Supplementary Table II. Primer for q-PCR.

**Gene target**	**Forward 5' → 3'**	**Reverse 5' → 3'**
L27	GCCAAGCGATCCAAGATCAA	GCTGGGTCCCTGAACACATC
Il10	CTTACTGACTGGCATGAGGATCA	GCAGCTCTAGGAGCATGTGG
Tgfb	ACCATGCCAACTTCTGTCTG	CGGGTTGTGTTGGTTGTAGA
Il17a	TTTTCAGCAAGGAATGTGGA	TCCATTGTGGAGGGCAGAC
Il22	ATGAGTTTTTCCCTTATGGGGAC	GCTGGAAGTTGGACACCTCAA
Il6	CACAGAGGATACCACTCCCAACA	TCCACGATTTCCCAGAGAACA
Il1b	AAGCCTCGTGCTGTCGGACC	TGAGGCCCAAGGCCACAGGT
Il23a	CGTATCCAGTGTGAAGATGGTT	CTATCAGGGAGTAGAGCAGGCT
Cxcl1	GACCATGGCTGGGATTCACC	GTGTGGCTATGACTTCGGTT
Cxcl5	TGCGTTGTGTTTGCTTAACCG	CTTCCACCGTAGGGCACTG
Reg3g	ATGCTTCCCCGTATAACCATCA	CGCTATTGAGCACAGATACGAG
Reg3b	ACTCCCTGAAGAATATACCCTCC	CGCTATTGAGCACAGATACGAG
16s rRNA	AGAGTTTGATCMTGGCTCAG	GGMTACCTTGTTACGACTT
Ccl22	TCTTGCTGTGGCAATTCAGA	GAGGGTGACGGATGTAGTCC
Aldh1a1	ATGGTTTAGCAGCAGGACTCTTC	CCAGACATCTTGAATCCACCGAA
Aldh1a2	GACTTGTAGCAGCTGTCTTCACT	TCACCCATTTCTCTCCCATTTCC
Aldh1a3	GAGCAGCAATTTCCTCCCATC	GAGCCGGTGAAGGCTATCT
Il22ra2	ACAACGAGCACTACTTTGTGCAG	CCCCCAGCAGTCAACTTTAT
Cd36	GCGACATGATTAATGGCACAG	GATCCGAACACAGCGTAGATAG
Il27ebi3	CAAGTACCGACTCCGCTACC	GGGTTTCGAGTTCCTGAGGG
Il27p28	GGCTATGTCCACAGCTTTGCT	CGAAGTGTGGTAGCGAGGAA
Ern1 exon 19-20	TGCTGAAACACCCCTTCTTC	GCCTCCTTTTCTATTCGGTCA
Xbp1s	AAGAACACGCTTGGGAATGG	CTGCACCTGCTGCGGAC
Duox2	TGCGCCTGTTACTGTGATTG	AATGGAAAGCAGCAGACAGC
Duoxa2	ACCGCTGCTCATTGTTATCC	AGTGCACAGCCACAATTTCG
Saa1/2	AGTGGCAAAGACCCCAATTA	GGCAGTCCAGGAGGTCTGTA
Muc2	ATGCCCACCTCCTCAAAGAC	GTAGTTTCCGTTGGAACAGTGAA
